# Suppressing the Electron–Phonon Coupling in 2D Perovskite Cs_3_Sb_2_I_9_ for Lead‐Free Indoor Photovoltaics

**DOI:** 10.1002/advs.202509281

**Published:** 2025-08-13

**Authors:** Yixin Guo, Fei Zhao, Chuanjun Zhang, Ping Wu, Jinchun Jiang, Jiahua Tao, Junhao Chu

**Affiliations:** ^1^ Mathematics and Science College Shanghai Normal University Shanghai 200234 China; ^2^ School of Photoelectric Engineering Changzhou Institute of Technology Changzhou Jiangsu 213002 China; ^3^ School of Mechanical Engineering Optoelectronics and Physics Huaihua University Huaihua 418000 China; ^4^ Engineering Research Center for Nanophotonics and Advanced Instrument Key Laboratory of Polar Materials and Devices Ministry of Education School of Physics and Electronic Science East China Normal University Shanghai 200241 China; ^5^ State Key Laboratory of Photovoltaic Science and Technology Institute of Optoelectronics Fudan University Shanghai 200438 China

**Keywords:** 2D C_3_Sb_2_I_9_, electron–phonon coupling, indoor photovoltaic, Pb‐free

## Abstract

Antimony‐based perovskite‐inspired materials (Sb‐PIMs) are promising lead‐free candidates for indoor photovoltaic application. Cs_3_Sb_2_I_9_, in particular, with a ≈2.0 eV bandgap, is ideal for harvesting indoor white light. However, solution‐processed Sb‐PIMs preferentially crystallize into thermodynamically stable 0D structures, leading to strong self‐trapped exciton (STE) formation, limiting device performance. Although chloride (Cl) doping can induce 2D structural transitions, it enhances Fröhlich electron–phonon coupling (EPC), creating an intrinsic trade‐off. Here, we develop an anion‐exchange strategy to fabricate phase‐pure, Cl‐free 2D Cs_3_Sb_2_I_9_ films that suppress STE formation while enabling controlled dimensional reconstruction. This approach yields a reduced Huang–Rhys factor (from 30.7 to 21.5) and prolonged STE lifetime (8.60 to 9.19 ps). Density functional theory (DFT) calculations reveal a significant reduction in excited‐state octahedral distortion (Δd = 0.898 × 10^−3^ for Cs_3_Sb_2_I_9_ vs. 5.752 × 10^−3^ for Cs_3_Sb_2_I_6_Cl_3_), confirming intrinsically weaker EPC in Cl‐free structures. The device achieves a power conversion efficiency (PCE) of 3.40% under AM 1.5G solar illumination and an 8.2% PCE under 1000 lux white LED conditions. alongside Long‐term stability measurement confirms its environmental robustness. These results represent the highest indoor performance reported to date for Sb‐based perovskite‐inspired solar cells.

## Graphical abstract

The study has developed an in situ anion‐exchange strategy to fabricate Pb‐free Sb‐based perovskite‐inspired materials (Sb‐PIMs) and solar cells, achieving a record indoor efficiency of 8.2% and a 3.4% power conversion efficiency under 1 sun illumination. Controlled structural dimensionality and suppressing Fröhlich electron–phonon coupling are realized through chloride‐free modulation, as evidenced by reduced Huang‐Rhys factors and DFT‐confirmed suppression of lattice distortions.

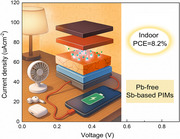

## 1 Introduction

With the rapid expansion of the Internet of Things (IoT), there is an increasing demand for self‐powered energy solutions tailored for distributed, low‐power electronic devices such as wireless sensors, health monitoring terminals, and wearable electronics. Indoor photovoltaic (IPV) technology, which harvests energy from ambient light sources, offers a sustainable and stable solution for powering these devices and has emerged as a key enabling technology for next‐generation intelligent systems.^[^ ^1^, ^2^, ^3^, ^4^ ^]^ Among the vast array of candidate materials, lead halide perovskites have demonstrated excellent indoor light harvesting performance due to their strong visible‐light absorption, tunable bandgaps, and cost‐effective fabrication processes, with power conversion efficiencies (PCEs) exceeding 40% under 1000 lux indoor illumination.^[^ ^4^, ^5^, ^6^, ^7^ ^]^ However, two major limitations hinder their practical application: the poor environmental stability of the photoactive layer under light, heat, humidity, and oxygen, and the inherent toxicity of lead, which restricts their safe integration into wearable and enclosed environments.^[^ ^8^, ^9^, ^10^, ^11^ ^]^

To address the challenges associated with the toxicity and stability of lead‐based perovskites, extensive research has focused on substituting Pb^2+^ with ns^2^‐type lone pair cations such as Sn^2+^, Sb^3+^, and Bi^3+^ to develop lead‐free perovskite and perovskite‐inspired materials.^[^ ^12^, ^13^, ^14^, ^15^ ^]^ Although Sn‐based perovskites exhibit similar crystal structures and ionic radii to Pb‐based counterparts, their high susceptibility to oxidation causes severe p‐type self‐doping, thereby limiting device performance.^[^ ^16^, ^17^, ^18^ ^]^ In contrast, antimony‐based perovskite‐inspired materials (A_3_Sb_2_I_9_, A = MA^+^ or Cs^+^) exhibit superior environmental stability and possess a direct bandgap of 1.9–2.05 eV, which aligns well with indoor lighting spectra, making them promising light‐harvesting materials for indoor applications.^[^ ^19^, ^20^, ^21^ ^]^ However, A_3_Sb_2_I_9_ compounds can form either 0D Sb_2_I_9_^3−^ dimer structures or 2D layered configurations.^[^ ^22^, ^23^, ^24^ ^]^ The 2D phase, featuring higher dielectric constants, improved carrier mobility, and narrower bandgaps, is more favorable for efficient photovoltaic operation.^[^ ^25^, ^26^, ^27^ ^]^ Nevertheless, due to the lower formation energy of the 0D phase, it tends to dominate during conventional solution processing, hindering the formation and utilization of the desirable 2D phase. For instance, Anupriya Singh et al. prepared 0D‐Cs_3_Sb_2_I_9_ films and modulated crystallization via Lewis base adducts, achieving a record PCE of 1.8% for 0D PIMs photovoltaic device under AM 1.5G illumination.^[^ ^28^ ^]^

To address this structural bottleneck, several strategies have been proposed to promote 2D phase formation and enhance device performance. For instance, Cai et al. introduced Cl^−^ into MA_3_Sb_2_I_9_ to modulate dimensionality, resulting in MA_3_Sb_2_I_9‐x_Cl_x_ films with low defect densities and stable I/Cl ratios. The devices achieved a PCE of 3.34% under AM 1.5G illumination, setting a new efficiency record for Sb‐based perovskite‐inspired solar cells.^[^ ^29^ ^]^ Xiao et al. reported that introducing methylammonium chloride (MACl) into Cs_3_Sb_2_I_9_ precursors under low‐pressure conditions facilitated the formation of intermediate structures consisting of coexisting 0D and 2D phases.^[^ ^30^ ^]^ During annealing, a complete 0D‐to‐2D phase transition occurred via I^−^/Cl^−^ exchange, leading to improved crystallinity and (201) preferred orientation, and delivering a PCE of 3.2%. Pecunia et al. further explored the potential of Sb‐based perovskite‐inspired materials in indoor photovoltaics by systematically evaluating BiOI and Cs_3_Sb_2_Cl_x_I_9‐x_ under typical indoor lighting conditions.^[^ ^31^ ^]^ Although both materials only achieved ≈1% efficiency under AM 1.5G solar illumination, their performance was significantly enhanced under fluorescent (FL) and white LED (WLED) sources, with PCEs reaching 4.4% for BiOI and 4.9% for Cs_3_Sb_2_Cl_x_I_9‐x_. These values rival or surpass those of commercial hydrogenated amorphous silicon (a‐Si:H) under indoor lighting. Moreover, the devices exhibited sufficient open‐circuit voltage (*V*_OC_) to power printed thin‐film transistor circuits, demonstrating their application potential in IoT power supplies and underscoring the feasibility of pnictohalide materials in distributed indoor energy systems.

Building upon these advances, Vivo et al. implemented a triple‐cation A‐site alloying strategy by co‐incorporating Cs^+^, MA^+^, and FA^+^ along with I^−^/Cl^−^ co‐doping at the X‐site to construct a compositionally engineered Cs_2.4_MA_0.5_FA_0.1_Sb_2_I_8.5_Cl_0.5_ system. This design maintained an optimal bandgap (1.9–2.0 eV), effectively suppressed defect‐assisted recombination, and achieved a record indoor PCE of 6.37%, the highest reported to date for Sb‐based halide photovoltaics.^[^ ^32^ ^]^ Despite these advancements, the efficiency of Sb‐based perovskite‐inspired solar cells remains well below their theoretical limit, with the underlying limiting mechanisms still not fully understood. Recent studies have suggested that strong electron–phonon coupling in Sb‐based materials induces self‐trapping of photoexcited carriers, which leads to severe non‐radiative recombination and limited carrier mobility.^[^ ^33^ ^]^ While this mechanism has been demonstrated in antimony chalcogenides, its manifestation and regulation in halide systems remain largely unexplored, particularly under structural dimensionality and halide doping. The above progress of Sb‐PIMs for photovoltaic application has been listed in **Table** **1**.

**Table 1:** The current progress of Sb‐PIMs for photovoltaic application.

Materials	Phase	Strategy	Performance	Reference
Cs_3_Sb_2_I_9_	0D	Lewis base adducts	1.8% (AM1.5G)	[28]
MA_3_Sb_2_I_9‐x_Cl_x_	2D	Cl doing	3.34% (AM1.5G)	[29]
Cs_3_Sb_2_Cl_x_I_9‐x_	2D	Cl doing	3.2% (AM1.5G)	[30]
Cs_3_Sb_2_Cl_x_I_9‐x_	2D	Cl doing	≈1% (AM1.5G) 4.9% (indoor) 4.9% (indoor)	[31]
Cs_2.4_MA_0.5_FA_0.1_Sb_2_I_8.5_Cl_0.5_	2D	Cl doing	2.47% (AM1.5G) 6.37% (indoor) 6.37% (indoor)	[32]
Cs_3_Sb_2_I_9_	2D	Cl doing and anion‐exchange	3.4% (AM1.5G) 8.2% (indoor)	**Our work**

In this work, we conduct a comprehensive spectroscopic and theoretical study to elucidate the nature of electron–phonon coupling in Cl‐doped 2D A_3_Sb_2_I_9‐x_Cl_x_ (A = MA^+^ or Cs^+^) films. While Cl doping facilitates 2D structure formation, we find that it simultaneously intensifies electron–phonon interactions, causing increased carrier localization and non‐radiative loss. To resolve this structure–coupling trade‐off, we propose an in situ anion exchange strategy to synthesize phase‐pure, Cl‐free 2D Cs_3_Sb_2_I_9_ films. This approach significantly enhances lattice rigidity and reduces the electron–phonon coupling strength by ≈30%, leading to improved carrier mobility and reduced recombination. As a result, carbon‐based planar devices incorporating this absorber achieved an indoor PCE of 8.2%, establishing a new benchmark for perovskite‐inspired indoor photovoltaics. This work provides new insights into the role of lattice dynamics in Sb‐PIMs and presents a viable route toward high‐efficiency, stable, and lead‐free indoor solar cells.

## 2 Results and Discussion

**Figure 1:**
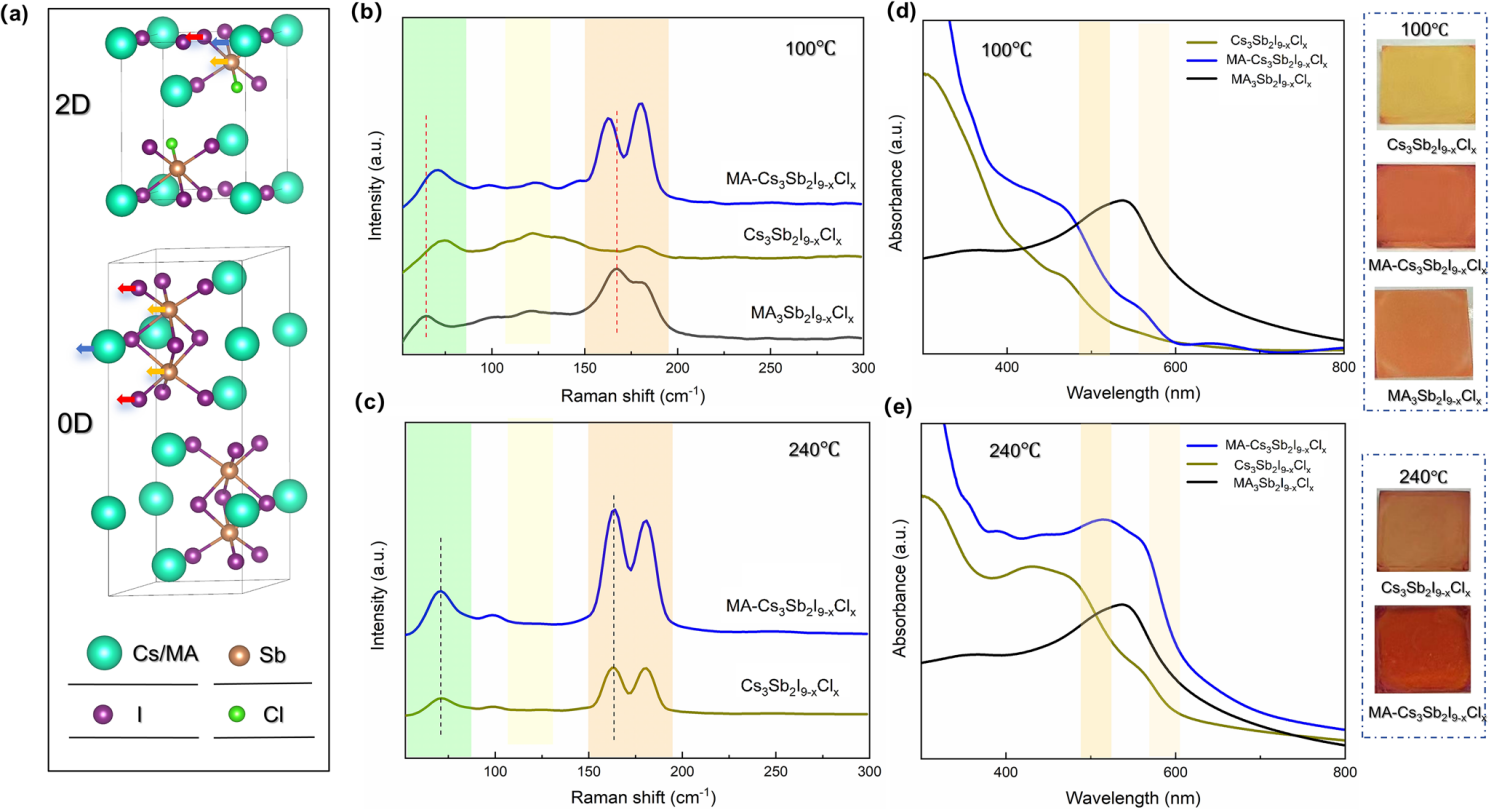
a) Crystal structures of typical 0D and 2D Sb‐based perovskite‐inspired materials. b) Raman spectra of MA_3_Sb_2_I_9‐x_Cl_x_, Cs_3_Sb_2_I_9‐x_Cl_x_, and MA‐Cs_3_Sb_2_I_9‐x_Cl_x_ films annealed at 100 °C. c) Raman spectra of Cs_3_Sb_2_I_9‐x_Cl_x_, and MA‐Cs_3_Sb_2_I_9‐x_Cl_x_ films annealed at 240 °C. d) Absorption spectra and optical images of MA_3_Sb_2_I_9‐x_Cl_x_, Cs_3_Sb_2_I_9‐x_Cl_x_, and MA‐Cs_3_Sb_2_I_9‐x_Cl_x_ films annealed at 100 °C. e) Absorption spectra and optical images of Cs_3_Sb_2_I_9‐x_Cl_x_, and MA‐Cs_3_Sb_2_I_9‐x_Cl_x_ films annealed at 240 °C, along with MA_3_Sb_2_I_9‐x_Cl_x_ film annealed at 100 °C.

Sb‐based perovskite‐inspired compounds crystallize in either 0D dimeric or 2D layered configurations, as depicted in **Figure** **1**a. These distinct dimensionalities strongly influence lattice dynamics and optoelectronic properties, making accurate structural identification essential for understanding performance‐limiting mechanisms. However, conventional X‐ray diffraction (XRD) provides limited phase discrimination due to severe peak overlap between 0D and 2D phases in the 2θ range of 20°–30°, particularly in mixed‐phase films.^[^ ^34^, ^35^ ^]^ To overcome this limitation, we employed Raman spectroscopy as a more dimension‐sensitive characterization technique. A phase‐pure 0D Cs_3_Sb_2_I_9_ reference was synthesized via hydrothermal methods,^[^ ^36^ ^]^ and its vibrational modes were assigned (Figure S1 and Table S1, Supporting Information).

Figure 1b presents the Raman spectra of MA_3_Sb_2_I_9‐x_Cl_x_, Cs_3_Sb_2_I_9‐x_Cl_x_, and MA‐Cs_3_Sb_2_I_9‐x_Cl_x_ films annealed at 100 °C. All samples exhibit broad low‐frequency bands (<75 cm^−1^), corresponding to [SbI_6_]^3−^ octahedral bending vibrations coupled with A‐site cations.^[^ ^37^ ^]^ A blueshift is observed with increasing Cs⁺ substitution, indicating enhanced lattice rigidity and higher structural symmetry.^[^ ^38^ ^]^ In the mid‐frequency region (100–150 cm^−1^), all spectra show broad stretching modes of [Sb_2_I_9_]^3−^ bioctahedra, confirming the persistence of 0D structures. A distinct vibrational mode at 163 cm^−1^, previously attributed to the 2D layered phase,^[^ ^37^ ^]^ appears only in MA‐containing films. Its absence in Cs_3_Sb_2_I_9‐x_Cl_x_ confirms that Cs‐dominated compositions retain the 0D structure after annealing at 100 °C, while the presence of MA⁺ drives a spontaneous dimensional transition to the 2D phase. These results highlight the critical role of A‐site cation composition in controlling dimensionality evolution and phase selection in Sb‐based halide systems.

To further correlate the structural dimensionality with optical characteristics, Figure 1d presents the absorption spectra of MA_3_Sb_2_I_9‐x_Cl_x_, Cs_3_Sb_2_I_9‐x_Cl_x_, and MA‐Cs_3_Sb_2_I_9‐x_Cl_x_ films annealed at 100 °C. Cs_3_Sb_2_I_9‐x_Cl_x_ films exhibit an absorption onset near 510 nm, corresponding to an optical bandgap of 2.42 eV (Figure S2, Supporting Information), which is consistent with the characteristic absorption of 0D [SbX_6_]^3−^ (X = I^−^/Cl^−^) isolated octahedra reported in literature.^[^ ^39^ ^]^ In contrast, the MA_3_Sb_2_I_9‐x_Cl_x_ films display a red‐shifted absorption edge ≈600 nm, yielding a narrower bandgap of 2.06 eV (Figure S2, Supporting Information), characteristic of 2D layered configurations composed of corner‐sharing [SbX_6_]^3−^ octahedra intercalated by MA⁺ cations.^[^ ^29^ ^]^ Notably, the MA‐Cs_3_Sb_2_I_9‐x_Cl_x_ hybrid films exhibit two distinct absorption edges near 510 and 600 nm, indicative of a mixed 0D/2D phase composition, consistent with the vibrational features observed in Raman spectra. To probe the thermally driven phase evolution, Raman spectra of Cs_3_Sb_2_I_9‐x_Cl_x_ and MA‐Cs_3_Sb_2_I_9‐x_Cl_x_ films were further collected after annealing at 240 °C, as shown in Figure 1c. At elevated temperature, the vibrational signatures attributed to the 0D phase, particularly the [SbI_6_]^3−^ stretching modes in the 80–150 cm^−1^ range, are significantly diminished in both Cs_3_Sb_2_I_9‐x_Cl_x_ and MA‐Cs_3_Sb_2_I_9‐x_Cl_x_ films. Meanwhile, modes associated with the 2D phase (150–175 cm^−1^) remain dominant, suggesting a thermally induced 0D‐to‐2D structural transition. The coexistence of dual absorption edges and the selective suppression of 0D Raman modes collectively confirm that high‐temperature annealing facilitates dimensional reconfiguration from 0D to 2D, particularly in the presence of partial MA⁺ incorporation.

Thermal annealing at elevated temperatures significantly influences both the optical response and crystalline order of Sb‐based perovskite‐inspired materials. As shown in Figure 1e, the absorption edge of the Cs_3_Sb_2_I_9‐x_Cl_x_ film shifts markedly from approximately 510 to 600 nm after annealing at 240 °C, indicative of a dimensional transition from 0D to 2D. In comparison, the MA‐Cs_3_Sb_2_I_9‐x_Cl_x_ film annealed at 240 °C exhibits a much steeper absorption onset ≈600 nm, along with a substantially enhanced absorbance in the 300–600 nm range, reflecting reduced lattice disorder, improved crystallinity, and enhanced light‐harvesting capability. These changes are further corroborated by the distinct film color transitions observed in the optical images (inset, Figure 1e), consistent with forming an ordered 2D structure.

The evolution of MA^+^ and Cl^−^ ions during thermal annealing was investigated via X‐ray photoelectron spectroscopy (XPS). As illustrated in the high‐resolution N 1s spectra (Figure S3, Supporting Information), the MA‐Cs_3_Sb_2_I_9‐x_Cl_x_ film annealed at 100 °C exhibits a distinct peak at ≈401.5 eV, characteristic of N component in MA^+^, confirming the retention of organic cations under low‐temperature annealing conditions. However, upon annealing at 240 °C, this MA^+^‐associated signal nearly vanishes. Similarly, the Cl 2p spectra (Figure S3, Supporting Information) reveal a pronounced reduction in Cl^−^ content for the MA‐containing film after 240 °C annealing, with the Cl 2p peak intensity dropping significantly compared to the 100 °C treated sample. This contrasts sharply with the behavior of the Cs_3_Sb_2_I_9‐x_Cl_x_ film (without MA^+^), where the Cl^−^ signal persists even at 240 °C, demonstrating the critical role of MA^+^ in enabling Cl^−^ removal. Therefore, the coupled elimination process can be conceptually summarized as:

(1) $$\begin{array}{c} \begin{array}{ccc} \mathrm{Sb}I_{3}+\mathrm{SbC}l_{3}+3\mathrm{MAI} & \overset{\;\;100^{\circ }C\;\;}{\to } & MA_{3}Sb_{2}I_{9-x}Cl_{x}\;(2D)\\ \mathrm{Sb}I_{3}+\mathrm{SbC}l_{3}+3\mathrm{CsI} & \overset{\;\;100^{\circ }C\;\;}{\to } & 0D(\mathrm{main})/2D\;\mathrm{phase}\\ & \overset{\;\;240^{\circ }C\;\;}{\to } & Cs_{3}Sb_{2}I_{9-x}Cl_{x}\;(2D)+\mathrm{SbC}l_{3}↑\\ 3\mathrm{MAI}+\mathrm{Sb}I_{3}+\mathrm{SbC}l_{3}+3\mathrm{CsI} & \overset{\;\;100^{\circ }C\;\;}{\to } & 2D(\mathrm{main})/0D\;\mathrm{phase}\\ & \overset{\;\;240^{\circ }C\;\;}{\to } & Cs_{3}Sb_{2}I_{9}\;(2D)+3\mathrm{MACl}\;↑ \end{array} \end{array}$$

To quantitatively demonstrate the thermodynamic preference for selective anion/cation exchange, we constructed a Cl‐ and MA‐contained 2 × 2 × 2 supercell of MACs_2_Sb_2_I_6_Cl_3_ and modeled its transformation into the Cl‐free Cs_3_Sb_2_I_9_ phase. Both MA and Cs sites were geometry‐optimized to identify the lowest‐energy configuration, as shown in Figure S4 (Supporting Information). The reaction enthalpies (ΔH) were calculated for the following two competitive pathways:

(2)

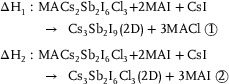

where ΔH = E[Cs_3_Sb_2_I_6_Cl_3_+3MAI or Cs_3_Sb_2_I_9_+3MACl]‐E[MACs_2_Sb_2_I_6_Cl_3_+2MAI+CsI], with total energies listed in Table S2 (Supporting Information). As shown in Figure S5 (Supporting Information), ΔH_1_ (reaction ①) is significantly lower than ΔH_2_ (reaction ②). According to the Brønsted–Evans–Polanyi (BEP) principle,^[^ ^40^, ^41^ ^]^ this implies that reaction ① proceeds more readily, indicating that Cl removal is thermodynamically favored. Furthermore, the negative ΔH confirms that Cl‐ and MA‐free 2D‐Cs_3_Sb_2_I_9_ is more thermodynamically stable than its Cl‐ and MA‐containing counterpart. A summary table (**Table** **2**) was provided to clarify the evolution of dimensionality in the dimensionality of MA_3_Sb_2_I_9‐x_Cl_x_, Cs_3_Sb_2_I_9‐x_Cl_x_, and MA‐Cs_3_Sb_2_I_9‐x_Cl_x_ films at 100 and 240 °C.

**Table 2:** The summary of the dimensionality changes for the three types of films annealed at 100 and 240 °C.

Sample	Annealing temperature	Phase‐Composition
MA_3_Sb_2_I_9‐x_Cl_x_ film	100 °C	2D‐MA_3_Sb_2_I_9‐x_Cl_x_ film
Cs_3_Sb_2_I_9‐x_Cl_x_ film	100 °C	0D(main)/2D‐intermediate film
MA‐Cs_3_Sb_2_I_9‐x_Cl_x_ film	100 °C	2D(main)/0D‐intermediate fim
Cs_3_Sb_2_I_9‐x_Cl_x_ film	240 °C	2D‐Cs_3_Sb_2_I_9‐x_Cl_x_ film
MA‐Cs_3_Sb_2_I_9‐x_Cl_x_ film	240 °C	2D‐Cs_3_Sb_2_I_9_ film

**Figure 2:**
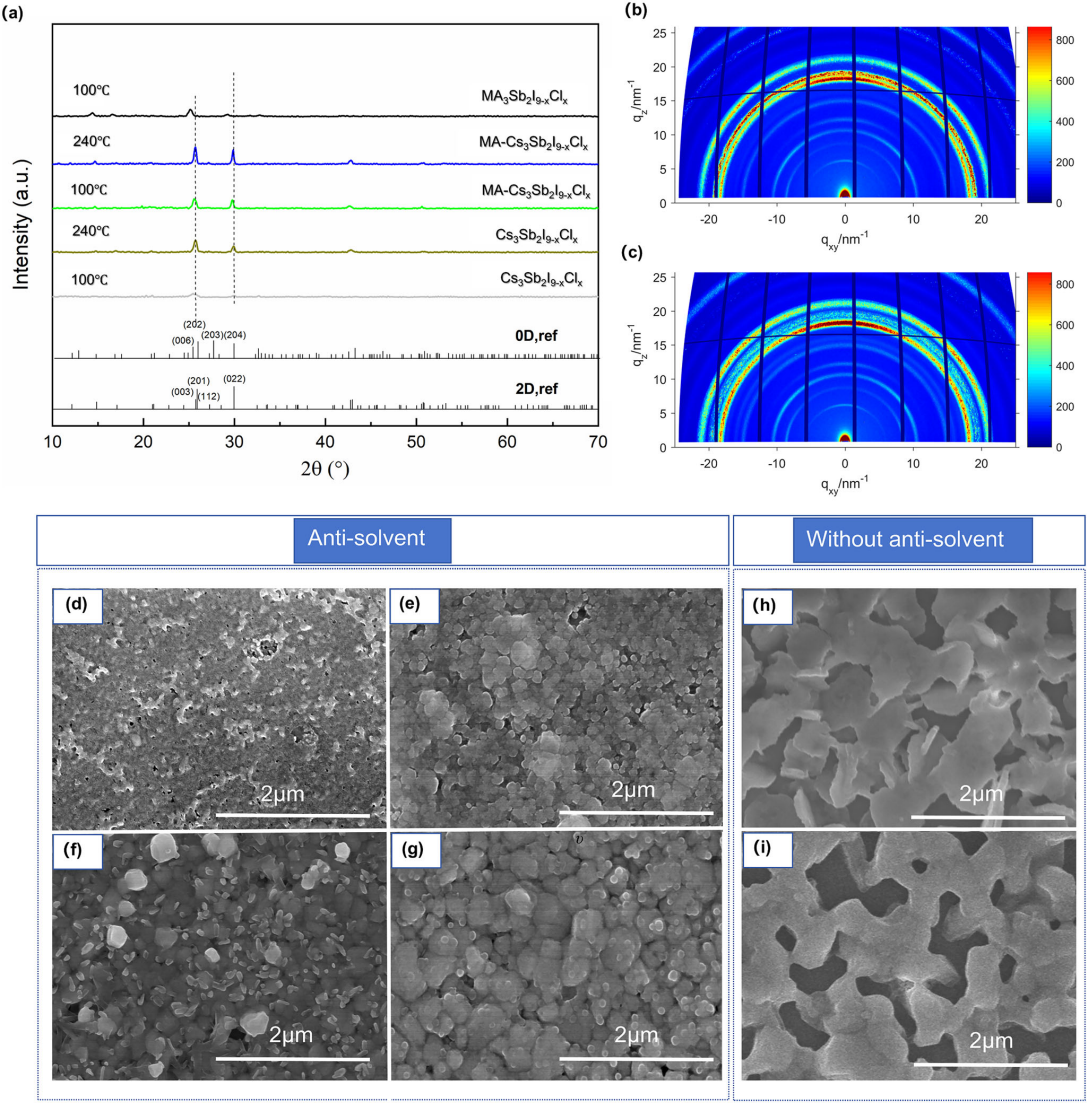
a) XRD patterns of MA_3_Sb_2_I_9‐x_Cl_x_, Cs_3_Sb_2_I_9‐x_Cl_x_ and MA‐Cs_3_Sb_2_I_9‐x_Cl_x_ annealed at 100 and 240 °C. b,c) GIWAX patterns of (b) Cs_3_Sb_2_I_9‐x_Cl_x_ and (c) MA‐Cs_3_Sb_2_I_9‐x_Cl_x_ films annealed at 240 °C. d–g) SEM images of Cs_3_Sb_2_I_9‐x_Cl_x_ and MA‐Cs_3_Sb_2_I_9‐x_Cl_x_ films treated with anti‐solvent and annealed at (d,e) 100 °C or (f,g) 240 °C. h,i) SEM images of Cs_3_Sb_2_I_9‐x_Cl_x_ and MA‐Cs_3_Sb_2_I_9‐x_Cl_x_ films annealed at 240 °C without anti‐solvent treatment.

The XRD patterns (**Figure** **2**a) also reveal structural evolution upon thermal annealing. For the 100 °C annealed samples, the Cs_3_Sb_2_I_9‐x_Cl_x_ film exhibits a weakly preferential (006) diffraction peak, indicative of the 0D dimer phase.^[^ ^42^ ^]^ In contrast, the XRD patterns of the MA‐Cs_3_Sb_2_I_9‐x_Cl_x_ film show a slight leftward shift, attributable to the larger ionic radius of MA^+^ (2.17Å) compared to Cs^+^ (1.67Å), along with two distinct peaks and reduced orientation preference. The appearance of a preferential (201) peak in MA_3_Sb_2_I_9‐x_Cl_x_ is characteristic of the 2D layered phase.^[^ ^4^ ^]^ Upon annealing at 240 °C, both Cs_3_Sb_2_I_9‐x_Cl_x_ and MA‐Cs_3_Sb_2_I_9‐x_Cl_x_ films transform predominantly into the 2D layered phase (space group *Pm1* (no. 164),^[^ ^43^ ^]^ consistent with Raman and optical measurements. Notably, the MA‐Cs_3_Sb_2_I_9‐x_Cl_x_ film (converted to 2D‐Cs_3_Sb_2_I_9_) displays more intense diffraction peaks and narrower full width at half maximum (FWHM), indicating enhanced crystallinity. This structural transformation suggests that the anion‐exchange method for obtaining Cl‐free Sb‐PIMs not only facilitates the dimensional transition from 0D to 2D but also modulates crystallization behaviour.

To gain further insights into crystallographic alignment, GIWAXS measurements were performed on the 2D‐Cs_3_Sb_2_I_9‐x_Cl_x_ and 2D‐Cs_3_Sb_2_I_9_ films (Figure 2b,c). The diffraction peak at q = 18.3 nm^−1^ corresponds to the (201) lattice plane, while those at q = 19.4 and 21.4 nm^−1^ are indexed to the (112) and (022) planes, respectively.^[^ ^30^, ^32^ ^]^ For 2D‐Cs_3_Sb_2_I_9‐x_Cl_x_, the dominant diffraction peak at q = 18.3 nm^−1^, indexed to the (201) plane, reveals a pronounced preferred orientation. The preferred orientation growth could result in direction‐dependent charge transport, which may limit vertical carrier extraction in devices. Conversely, 2D‐Cs_3_Sb_2_I_9_ displays a greater azimuthal intensity distribution, indicating a randomized in‐plane grain orientation. This randomized orientation mitigates in‐plane anisotropy, promoting vertically aligned pathways that enable more balanced charge transport and reduced recombination losses.^[^ ^44^ ^]^

To establish a structure‐morphology relationship, scanning electron microscopy (SEM) was performed on the same set of samples (Figure 2d–i). For films annealed at 100 °C (Figure 2d,e), the MA‐contained film exhibits significantly larger grains and superior surface coverage than its Cs‐only counterpart, consistent with the XRD results. This could be attributed to the templating effect of MA^+^, which lowers the nucleation barrier for the 2D intermediate film and facilitates lateral grain coalescence during crystallization, resulting in enhanced film uniformity. Upon annealing at 240 °C (Figure 2f,g), the MA‐Cs_3_Sb_2_I_9‐x_Cl_x_ film (converted to 2D‐Cs_3_Sb_2_I_9_ film) shows a marked increase in grain size, improved surface smoothness, and reduced grain boundary density. In contrast, although the grain size of the 2D‐Cs_3_Sb_2_I_9‐x_Cl_x_ film also increases after annealing, the surface remains relatively rough and exhibits noticeable pinholes. Control films were fabricated without anti‐solvent treatment to isolate the role of anti‐solvent processing. Without anti‐solvent treatment, the Cs_3_Sb_2_I_9‐x_Cl_x_ film exhibits a porous morphology with disordered hexagonal nanoplatelets (Figure 2h) due to the absence of rapid crystallization induced by anti‐solvent. In contrast, the MA‐Cs_3_Sb_2_I_9‐x_Cl_x_ film, although still showing some porosity, consists of irregularly shaped grains instead of large hexagonal nanoplatelets (Figure 2i). This morphological difference confirms that MA⁺ can reduce the nucleation barrier and promote a more uniform nucleation process. However, without an anti‐solvent, the overall crystallization rate remains slow, limiting the formation of a fully compact film and resulting in residual porosity. Therefore, using an anti‐solvent is essential to achieve ideal film morphology. These morphological analysis results demonstrate that MA^+^ facilitates phase evolution toward a stable Cl‐free 2D structure and regulates crystallization kinetics, balancing nucleation and growth.

**Figure 3:**
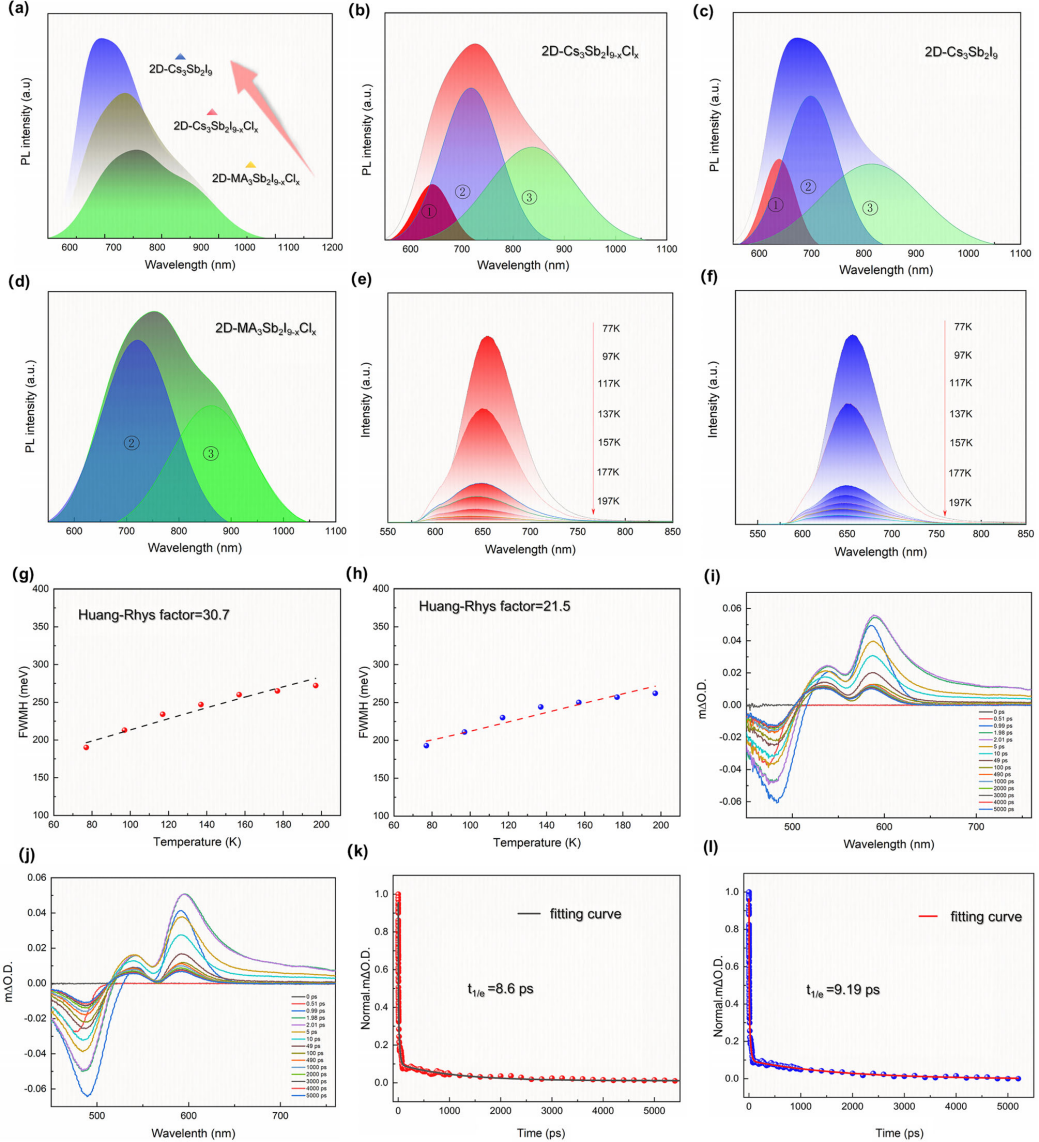
a) Room temperature PL spectra of 2D‐MA_3_Sb_2_I_9‐x_Cl_x_, 2D‐Cs_3_Sb_2_I_9‐x_Cl_x_, and 2D‐Cs_3_Sb_2_I_9_ films. b‐d) Gaussian deconvolution of PL spectra for (b) 2D‐Cs_3_Sb_2_I_9‐x_Cl_x_, (c) 2D‐Cs_3_Sb_2_I_9_, and (d) 2D‐MA_3_Sb_2_I_9‐x_Cl_x_ films. e,f) Temperature‐dependent PL spectra of (e) 2D‐Cs_3_Sb_2_I_9‐x_Cl_x_ and (f) 2D‐Cs_3_Sb_2_I_9_ films. g,h) FWHM as a function of temperature extracted from (g) 2D‐Cs_3_Sb_2_I_9‐x_Cl_x_ and (h) 2D‐Cs_3_Sb_2_I_9_ films. i,j) TA spectra at different time delays for (i) Cs_3_Sb_2_I_9‐x_Cl_x_ and (j) 2D‐Cs_3_Sb_2_I_9_ films. k,l) TA decays of (k) glass/2D‐Cs_3_Sb_2_I_9‐x_Cl_x_ and (l) glass/2D‐Cs_3_Sb_2_I_9_ films probed at 600 nm.

To gain mechanistic insight into the optoelectronic behavior of Sb‐PIMs, steady‐state and time‐resolved spectroscopic studies were conducted. Room‐temperature photo luminescence (PL) spectra (**Figure** **3**a) reveal a broad emission band centered at ≈750 nm for 2D‐MA_3_Sb_2_I_9‐x_Cl_x_, corresponding to a sizable Stokes shift (≈0.4 eV), indicative of pronounced electron–phonon coupling and strong carrier localization that favors the formation of self‐trapped excitons (STEs).^[^ ^45^ ^]^ In contrast, 2D‐Cs_3_Sb_2_I_9‐x_Cl_x_ and 2D‐Cs_3_Sb_2_I_9_ exhibit progressively narrower PL profiles, with red‐shifted emission maxima and reduced Stokes shifts, suggesting suppressed lattice distortion and weakened self‐trapping tendencies. The normalized PL spectra were deconvoluted to resolve the underlying recombination channels using Gaussian fitting (Figure 3b–d).

As reported,^[^ ^34^, ^46^ ^]^ the PL spectrum of 2D‐Cs_3_Sb_2_I_9_ comprises three distinct components: a short‐wavelength peak assigned to band‐to‐band emission ①, a mid‐wavelength feature arising from STE emission ②, and a long‐wavelength shoulder related to defect‐assisted recombination ③. 2D‐MA_3_Sb_2_I_9‐x_Cl_x_ exhibits only two dominant peaks, consistent with its strong structural distortion and enhanced charge localization, which inhibits direct band‐edge transitions. In comparison, 2D‐Cs_3_Sb_2_I_9_ displays significantly reduced STE‐ and defect‐related contributions, indicative of diminished nonradiative pathways and lower defect density.^[^ ^47^ ^]^ The schematic of the photophysical process for PL spectra is shown in Figure S6 (Supporting Information).

To further investigate the exciton‐phonon coupling strength, temperature‐dependent PL spectra were recorded, as presented in Figure 3e,f. Both films exhibit spectral narrowing at low temperatures. The FWHM values were fitted using the standard phonon broadening model:

(3) $$\mathrm{FWHM}(T)=2.36\sqrt{S}\hbar w_{ph}\sqrt{\coth\frac{\hbar w_{ph}}{2k_{b}T}}$$

here, ℏ*w*_ph_ is the phonon frequency, *k_b_* denotes the Boltzmann constant, and S is the Huang‐Rhys factor. The fitting S factor can be observed in Figure 3g,h. The extracted S values decrease from 30.7 in 2D‐Cs_3_Sb_2_I_9‐x_Cl_x_ to 21.5 in 2D‐Cs_3_Sb_2_I_9_, confirming a substantial reduction in electron–phonon coupling and improved structural rigidity upon Cl removal.

To better understand the dynamics of photogenerated carriers and gain mechanistic insights into the excited‐state relaxation pathways, femtosecond transient absorption (TA) spectroscopy was performed for both 2D‐Cs_3_Sb_2_I_9‐x_Cl_x_ and 2D‐Cs_3_Sb_2_I_9_ films (Figure 3i,j). Upon photoexcitation, a prominent photo‐bleaching (PB) signal centered around 480 nm is observed, which originates from excitonic ground‐state bleaching and marks the optical transition resonance. Concurrently, two photoinduced absorption (PIA) bands emerge at approximately 530 and 600 nm, in good agreement with previously reported signatures for self‐trapped excitonic states in Sb‐based halide systems.^[^ ^32^ ^]^ These features are ascribed to a Stark‐effect‐induced redshift and broadening of the exciton absorption profile, reflecting strong exciton–lattice interactions and dynamic carrier localization.^[^ ^48^ ^]^ To elucidate the excited‐state relaxation dynamics, transient absorption (TA) spectroscopy was conducted on both 2D‐Cs_3_Sb_2_I_9‐x_Cl_x_ and 2D‐Cs_3_Sb_2_I_9_ films, with the decay kinetics monitored at 600 nm and fitted using a tri‐exponential model (Figure 3k,l; Table S3, Supporting Information). The decay components can be attributed to three physical processes: an ultrafast component (τ₁) representing initial carrier trapping, a mid‐fast component (τ_2_) related to nonradiative recombination, and a long‐lived tail (τ_3_) associated with radiative recombination of self‐trapped excitons (STEs).^[^ ^49^ ^]^

(4) $$I(\;t)=A_{1}\;e^{-t/\tau_{1}}+A_{2}e^{-t/\tau_{2}}+A_{3}e^{-t/\tau_{3}}$$

Quantitatively, the 2D‐Cs_3_Sb_2_I_9_ film exhibits prolonged lifetimes of τ₁ = 2.45 ps, τ_2_ = 24.49 ps, and a long‐lived τ_3_ = 1737.6 ps, resulting in an effective decay time (τ₁/e) of 9.19 ps. In contrast, 2D‐Cs_3_Sb_2_I_9‐x_Cl_x_ shows shorter corresponding lifetimes of τ₁ = 2.01 ps, τ_2_ = 23.12 ps, τ_3_ = 1078.4 ps, and τ₁/e = 8.6 ps. These results indicate that the Cl‐free 2D‐Cs_3_Sb_2_I_9_ system effectively suppresses ultrafast trapping and nonradiative loss channels, while promoting radiative recombination pathways. The prolonged τ_3_ and τ₁/e values strongly suggest reduced self‐trapping behavior and enhanced excited‐state stability, in line with the observed improvements in PL spectral features and photovoltaic efficiency.

**Figure 4:**
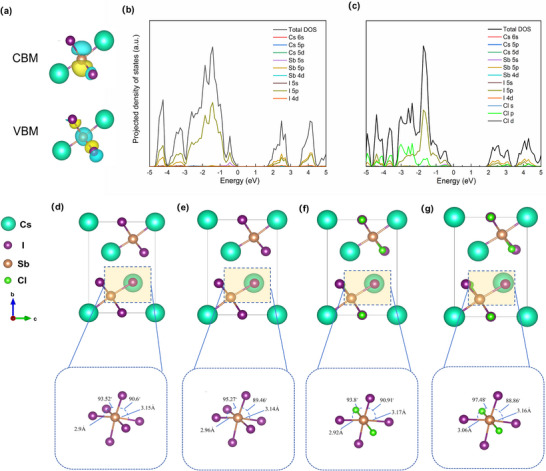
a) Charge density plots of the VBM and CBM for 2D‐Cs_3_Sb_2_I_9_. b,c) PDOS for (b) 2D‐Cs_3_Sb_2_I_9_ and (c) 2D‐Cs_3_Sb_2_I_6_Cl_3_. d,e) Ground‐state optimized structures from DFT calculations for (d) 2D‐Cs_3_Sb_2_I_9_ and (e) 2D‐Cs_3_Sb_2_I_6_Cl_3_. f,g) Relaxed excited‐state geometries for (f) 2D‐Cs_3_Sb_2_I_9_ and (g) 2D‐Cs_3_Sb_2_I_6_Cl_3_.

Recent studies have demonstrated the critical role of excited‐state lattice distortion in governing the magnitude of Stokes shift and the propensity for self‐trapped exciton (STE) formation, particularly in low‐dimensional halide systems.^[^ ^50^, ^51^, ^52^ ^]^ To elucidate the structural–electronic coupling mechanisms underpinning this behavior, we conducted first‐principles density functional theory (DFT) calculations to investigate the electronic structures and excited‐state geometries of 2D‐Cs_3_Sb_2_I_9_ and 2D‐Cs_3_Sb_2_I_6_Cl_3_. As shown in **Figure** **4**a, the charge density and projected density of states (PDOS, Figure 4b) for 2D‐Cs_3_Sb_2_I_9_ reveal that the valence band maximum (VBM) is primarily composed of Sb 5s and I 5p orbitals, while the conduction band minimum (CBM) is dominated by hybridized Sb 5p and I 5p orbitals, confirming that photoexcitation predominantly involves transitions within the [SbI_6_]^3−^ octahedra. In contrast, Cl atoms in 2D‐Cs_3_Sb_2_I_6_Cl_3_ (x = 3) do not directly contribute to the VBM or CBM (Figure 4c), but instead modulate the local electronic environment by altering the geometry of the [SbX_6_]^3−^ coordination units, thereby indirectly affecting the electronic structure.

To evaluate structural evolution under photoexcitation, ground‐ and excited‐state geometries were optimized (Figure 4d–g). In the excited state, 2D‐Cs_3_Sb_2_I_6_Cl_3_ exhibits pronounced bond length asymmetry and octahedral distortion relative to its Cl‐free counterpart,^[^ ^53^, ^54^ ^]^ consistent with its stronger electron–phonon coupling and enhanced structural relaxation, as corroborated by broader PL emission and shorter carrier lifetimes observed experimentally. The extent of lattice distortion was further quantified using the distortion index (Δd), defined as:^[^ ^55^ ^]^

(5) $$\Delta d=\left(\frac{1}{n}\right){\sum_{i=1}^{n}\left[\frac{d_{n}-d_{ave}}{d_{ave}}\right]}^{2}$$

where *d*_n_ denotes individual Sb–X bond lengths and *d*_ave_ is their average. In 2D‐Cs_3_Sb_2_I_9_, the Sb–I bonds are grouped into three identical lengths of 3.140 Å and three of 2.958 Å, yielding a nearly symmetric coordination geometry. Conversely, 2D‐Cs_3_Sb_2_I_6_Cl_3_ exhibits two short Sb–Cl bonds (2.506 and 2.908 Å) and four asymmetric Sb–I bonds (3.061 and 3.157 Å), indicative of substantial octahedral distortion. As a result, the *Δd* value rises from 0.898 × 10^−3^ in the Cl‐free structure to 5.752 × 10^−3^ upon Cl incorporation (Table S4, Supporting Information), marking a nearly one order of magnitude increase in lattice distortion. These findings demonstrate that Cl substitution, while not directly modifying band‐edge states, perturbs the excited‐state lattice geometry and amplifies the propensity for STE formation via enhanced electron–phonon coupling. In contrast, the reduced distortion in 2D‐Cs_3_Sb_2_I_9_ leads to suppressed carrier self‐trapping and improved transport characteristics, thereby underpinning its superior optoelectronic performance in indoor photovoltaics.

**Figure 5:**
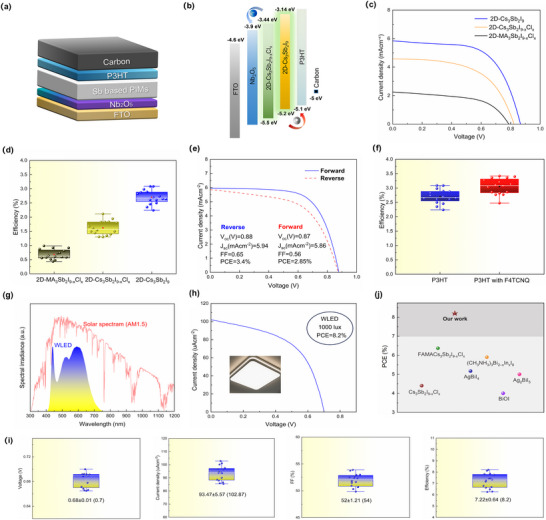
Device configuration and photovoltaic performance of Sb‐based perovskite‐inspired solar cells. a) Schematic of the planar architecture: FTO/Nb_2_O_5_/PIMs/P3HT (or F4TCNQ:P3HT)/Carbon. b) Energy level alignment of each functional layer. c) *J–V* curves of 2D‐MA_3_Sb_2_I_9‐x_Cl_x_, 2D‐Cs_3_Sb_2_I_9‐x_Cl_x_, and 2D‐Cs_3_Sb_2_I_9_ devices under AM1.5G. d) Efficiency statistics of corresponding devices. e) *J–V* curves of 2D‐Cs_3_Sb_2_I_9_ devices with pristine or F4TCNQ‐doped P3HT HTL. f) Efficiency distributions with and without F4TCNQ doping. g) Comparison of AM1.5G and WLED spectra. h) *J–V* curve of 2D‐Cs_3_Sb_2_I_9_ device under 1000 lux WLED. i) Indoor PCE statistics under WLED. j) PCE comparison with reported Sb/Bi‐based perovskite‐inspired indoor photovoltaics.

To validate the optoelectronic performance of the developed materials under both outdoor and indoor illumination, planar heterojunction devices with the structure FTO/Nb_2_O_5_/absorber /P3HT (or F4TCNQ‐doped P3HT)/carbon were fabricated (**Figure** **5**a). Figure 5b exhibits the relevant energy level diagram of the corresponding devices. The CBM and VBM of 2D‐Cs_3_Sb_2_Cl_x_I_9‐x_ and 2D‐Cs_3_Sb_2_I_9_ film can be calculated by using Fermi edges, secondary electron cutoff edges (Figure S7, Supporting Information), and bandgaps (Figure S8, Supporting Information).^[^ ^56^ ^]^ Compared to its Cl‐contained counterpart, the VBM of Cl‐free Cs_3_Sb_2_I_9_ is much closer to that of P3HT, facilitating more efficient hole extraction.^[^ ^57^, ^58^ ^]^ Under standard AM 1.5G solar illumination, devices based on 2D‐MA_3_Sb_2_I_9‐x_Cl_x_, 2D‐Cs_3_Sb_2_I_9‐x_Cl_x_, and Cl‐free 2D‐Cs_3_Sb_2_I_9_ films were evaluated as absorber layers. Figure 5c and Table S5 (Supporting Information) show that device performance improves with increasing crystallographic dimensionality from 0D to 2D. Specifically, the 2D‐MA_3_Sb_2_I_9‐x_Cl_x_ device achieved a PCE of 0.98% with *V*_OC_ = 0.79 V, *J*_SC_ = 2.26 mA·cm^−2^, and FF = 0.55. The 2D‐Cs_3_Sb_2_I_9‐x_Cl_x_ device further improved to a PCE of 1.47%, *V*_OC_ = 0.82 V, *J*_SC_ = 4.58 mA·cm^−2^, and FF = 0.56. Remarkably, the Cl‐free 2D‐Cs_3_Sb_2_I_9_ device delivered a substantially enhanced performance with a PCE of 3.09%, *V*_OC_ = 0.87 V, *J*_SC_ = 5.83 mA·cm^−2^, and FF = 0.61, representing more than a twofold efficiency gain over Cl‐doped analogs. The statistical device distributions (Figure 5d; Figure S9, Supporting Information) further verify the robustness and reproducibility of this dimensional tuning strategy. Moreover, the external quantum efficiency (EQE) spectrum of the 2D‐Cs_3_Sb_2_I_9_ device (Figure S10, Supporting Information) demonstrates excellent correlation with its *J–V* characteristics, yielding an integrated *J*_SC_ of 5.89 mA·cm^−2^, which is in close agreement with the value obtained from *J–V* measurements. This consistency substantiates the intrinsic photogeneration capability of the device and affirms the accuracy of its broadband photoresponse under AM 1.5G conditions.To further enhance hole extraction and optimize interfacial energetics, the strong electron acceptor molecule F4TCNQ was introduced to dope the P3HT hole transport layer.^[^ ^59^, ^60^ ^]^ After optimization, the doped device exhibited a PCE of 3.40% (an ≈10% improvement), with *V*_OC_ = 0.87 V, *J*_SC_ = 5.94 mA·cm^−2^, and FF = 0.65 (Figure 5e; Table S6, Supporting Information). The statistical device distributions (Figure S11, Supporting Information) further confirm that devices employing F4TCNQ‐doped P3HT exhibit higher average PCE (3.05 ± 0.28%) compared to the undoped counterparts (2.7 ± 0.25%). The significantly suppressed hysteresis (Figure 5e) and narrower performance spread (Figure 5f) confirm that F4TCNQ doping promotes hole transport, facilitates interfacial charge extraction, and mitigates recombination losses.^[^ ^61^ ^]^

Under simulated indoor conditions (WLED, 1000 lux, 3500 K), the 2D‐Cs_3_Sb_2_I_9_ device exhibited exceptional photovoltaic performance (Figure 5g,h), achieving a record indoor PCE of 8.2% with *J*_SC_ = 102.87 µA·cm^−2^, *V*_OC_ = 0.70 V, and FF = 0.52. The averaged metrics across multiple devices yielded PCE = 7.22 ± 0.64%, *J*_SC_ = 93.47 ± 5.57 µA·cm^−2^, *V*_OC_ = 0.68 ± 0.01 V, and FF = 0.52 ± 0.01, outperforming all previously reported Sb/Bi‐based indoor photovoltaic systems (Figure 5j; Table S7, Supporting Information). Benefiting from the synergistic combination of in situ anion exchange and A‐site compositional regulation, the resulting 2D Cs_3_Sb_2_I_9_ absorber layer exhibited suppressed electron–phonon coupling and reduced self‐trapped exciton formation. When coupled with F4TCNQ‐doped interface engineering, this led to enhanced charge extraction and minimized recombination losses. The final device thus achieves benchmark PCEs of 3.40% under AM 1.5G and 8.2% under WLED illumination, representing a new performance milestone for Sb‐based indoor photovoltaics. This work establishes a generalizable materials and design strategy toward efficient, stable, and lead‐free indoor photovoltaic technologies. Moreover, there remains substantial potential for further PCE enhancement through systematic optimization of the electron transport layer and hole transport layer architectures for charge mobility improvements in the future.^[^ ^62^ ^]^ To assess long‐term operational stability, we monitored unencapsulated 2D‐Cs_3_Sb_2_I_9_ devices under ambient atmosphere with a relative humidity (RH) of ≈40% at room temperature. As shown in Figure S12 (Supporting Information), the normalized PCE retained 90% of its initial value after 120 hours of exposure. The average device performance and corresponding standard deviations are presented to ensure statistical reliability.

## 3 Conclusion

In this work, we resolve a long‐overlooked contradiction between chloride‐induced dimensional modulation and the concurrent enhancement of EPC in Sb‐PIMs. By integrating systematic spectroscopic characterization with first‐principles calculations, we report an anion‐exchange strategy that enables the formation of Cl‐free 2D Cs_3_Sb_2_I_9_ films while fundamentally suppressing EPC‐driven carrier self‐trapping. Quantitative analysis reveals a reduction in the Huang–Rhys factor (from 30.7 to 21.5) and a prolonged STE lifetime (from 8.60 to 9.19 ps). Density functional theory confirms a markedly lower octahedral distortion in the excited state (*Δd* = 0.898 × 10^−3^ vs 5.752 × 10^−3^), affirming suppressed exciton localization. At the device level, planar heterojunction solar cells employing this Cl‐free 2D structure and F4TCNQ‐doped interfacial engineering achieve record efficiencies of 3.40% under AM 1.5G and 8.2% under indoor WLED lighting, setting new benchmarks for Sb‐based PIM photovoltaics. Beyond these performance advances, this work establishes a robust structure, property, device correlation, offering a versatile design framework for modulating EPC in low‐dimensional semiconductors and advancing the development of stable, lead‐free indoor photovoltaic technologies.

## 4 Experimental Section

### Device Fabrication

Fluorine‐doped tin oxide (FTO) substrates were first patterned via chemical etching and subsequently cleaned by sequential ultrasonic treatment in soap solution, deionized water, and ethanol. The substrates were then dried under a stream of high‐purity nitrogen. A 100 nm Nb_2_O_5_ compact layer was deposited by high‐vacuum sputtering (Angstrom Engineering) and thermally annealed at 500 °C for 30 minutes. The precursor solution for MA‐Cs_3_Sb_2_I_9‐x_Cl_x_ was prepared by dissolving 0.75 m CsI (Sigma‐Aldrich, 99.9%), 0.75 M MAI (Xi'an Polymer Light Technology Corp., 99.99%), 0.25 m SbI_3_ (Sigma‐Aldrich, 98%), and 0.3125 M SbCl_3_ (Sigma‐Aldrich, 99.95%) in anhydrous DMF (Sigma‐Aldrich, 99.8%). To selectively fabricate MA_3_Sb_2_I_9‐x_Cl_x_ or Cs_3_Sb_2_I_9‐x_Cl_x_ compositions, MAI or CsI was respectively excluded from the precursor formulation. The precursor was spin‐coated onto the Nb_2_O_5_‐coated FTO substrates at 3000 rpm for 30 seconds, with anisole applied as an anti‐solvent during the process. Post‐deposition annealing was performed via SbI_3_ vapor‐assisted treatment at 100 or 240 °C for 10 minutes.^[^ ^46^ ^]^ For the hole transport layer (HTL), a 6 mg mL^−1^ solution of P3HT (Sigma‐Aldrich) in chlorobenzene, containing 1 wt.% F4‐TCNQ (Sigma‐Aldrich, 97%), was spin‐coated at 3000 rpm, followed by thermal annealing at 100 °C for 5 minutes. After cooling to room temperature, commercial carbon paste was screen‐printed onto the HTL and dried at 120 °C on a hot plate for 15 minutes.

### Characterization

X‐ray diffraction (XRD) patterns were collected using a Bruker D8 Advance powder diffractometer. Grazing‐incidence wide‐angle X‐ray scattering (GIWAXS) measurements were performed at the BL14B1 beamline of the Shanghai Synchrotron Radiation Facility (SSRF). X‐ray photoelectron spectroscopy (XPS) and ultraviolet photoelectron spectroscopy (UPS) were conducted using a Thermo Scientific ESCALAB 250Xi system, equipped with a He I (21.2 eV) UV discharge source for UPS analysis. The optical absorption spectra were recorded using a Cary 5000 UV–vis–NIR spectrophotometer. Transient absorption spectroscopy (TAS) measurements were carried out using a HELIOS femtosecond pump–probe system. Surface morphology and microstructure were characterized by scanning electron microscopy (SEM) using a HITACHI S‐4800 instrument. Room‐temperature Raman and photoluminescence (PL) spectra were obtained using a HORIBA LabRAM spectrometer with 785 and 532 nm excitation lasers, respectively. Current density‐voltage (*J–V*) measurements were conducted using a Keithley 2400 source meter under simulated AM 1.5G illumination (100 mW cm^−2^) from a Newport Oriel Sol3A solar simulator, calibrated with a certified silicon reference cell (Newport Oriel 91 150 V, active area: 0.09 cm^2^). The incident photon‐to‐current conversion efficiency (IPCE) spectra were recorded using a QTEST 1000AD station equipped with a calibrated reference silicon cell. Indoor *J–V* characteristics were measured under a 3500 K white LED (WLED) light source (Philips) positioned to provide an illuminance of 1000 lux, as verified with a TES 1332A digital lux meter. The corresponding power density was determined using an LX‐107 optical power meter.

### Computational Part

Density functional theory (DFT) simulations were carried out using the Vienna Ab initio Simulation Package (VASP). The structural optimizations were further performed by VASP until the total energy converged to within 10^−6^ eV. The primary geometry‐optimized for supercells was performed by the DS‐PAW software to accelerate the convergence. The exchange‐correlation potential was described by the generalized gradient approximation (GGA) using the HSE06 hybrid functional. A Monkhorst–Pack k‐point mesh of 5 × 5 × 3 was employed for the density of states (DOS) calculations. To obtain the excited‐state geometry, the procedure was as follows: First, the ground‐state structure was fully optimized under standard relaxation settings. Subsequently, the EIGENVAL file from a static calculation was examined to assess the occupation of specific electronic states. The spin‐resolved occupation was then manually adjusted using the FERWE and FERDO tags to enforce desired electronic occupations. Finally, the excited‐state structure was obtained by relaxing the geometry under the constrained occupation setup.

## Conflict of Interest

The authors declare no conflict of interest.

## Supporting information



Supporting Information

## Data Availability

The data that support the findings of this study are available from the corresponding author upon reasonable request.
