# Supplementary material for: Suppressing the Electron–Phonon Coupling in 2D Perovskite Cs3Sb2I9 for Lead‐Free Indoor Photovoltaics
Source: Adv Sci (Weinh). 2025 Aug 13;12(41):e09281. doi: 10.1002/advs.202509281 (PMC12591184; doi:10.1002/advs.202509281)
Supplement: Supplementary file 1 — Supporting Information [file ADVS-12-e09281-s001.docx]

Copyright WILEY-VCH Verlag GmbH & Co. KGaA, 69469 Weinheim, Germany, 2013.

Supporting Information

**Suppressing the Electron-Phonon Coupling** **in 2D** **Perovskite Cs_3_Sb_2_I_9_ for Lead-Free Indoor Photovoltaics**

*Yixin Guo, Fei Zhao*, Chuanjun Zhang, Ping Wu, Jinchun Jiang, Jiahua Tao*, Junhao Chu*

Dr. Y. Guo, Dr. C. Zhang

Mathematics and Science College, Shanghai Normal University, Shanghai 200234, China

Dr. F. Zhao

School of Photoelectric Engineering, Changzhou Institute of Technology, Changzhou, Jiangsu, 213002, China

1. mail: zhaofei@czu.cn

Dr. Ping Wu

School of Mechanical Engineering, Optoelectronics and Physics, Huaihua University, Huaihua, 418000, China

Prof. J. Jiang, Prof. J. Chu, Prof. J. Tao

Engineering Research Center for Nanophotonics and Advanced Instrument, Key Laboratory of Polar Materials and Devices, Ministry of Education, School of Physics and Electronic Science, East China Normal University, Shanghai 200241, China

E-mail: jhtao@phy.ecnu.edu.cn

Prof. J. Chu

State Key Laboratory of Photovoltaic Science and Technology, Institute of Optoelectronics, Fudan University, Shanghai, China





**Figure S1**. Raman images of 0D-Cs_3_Sb_2_I_9_ power and 2D-Cs_3_Sb_2_I_9_ film.


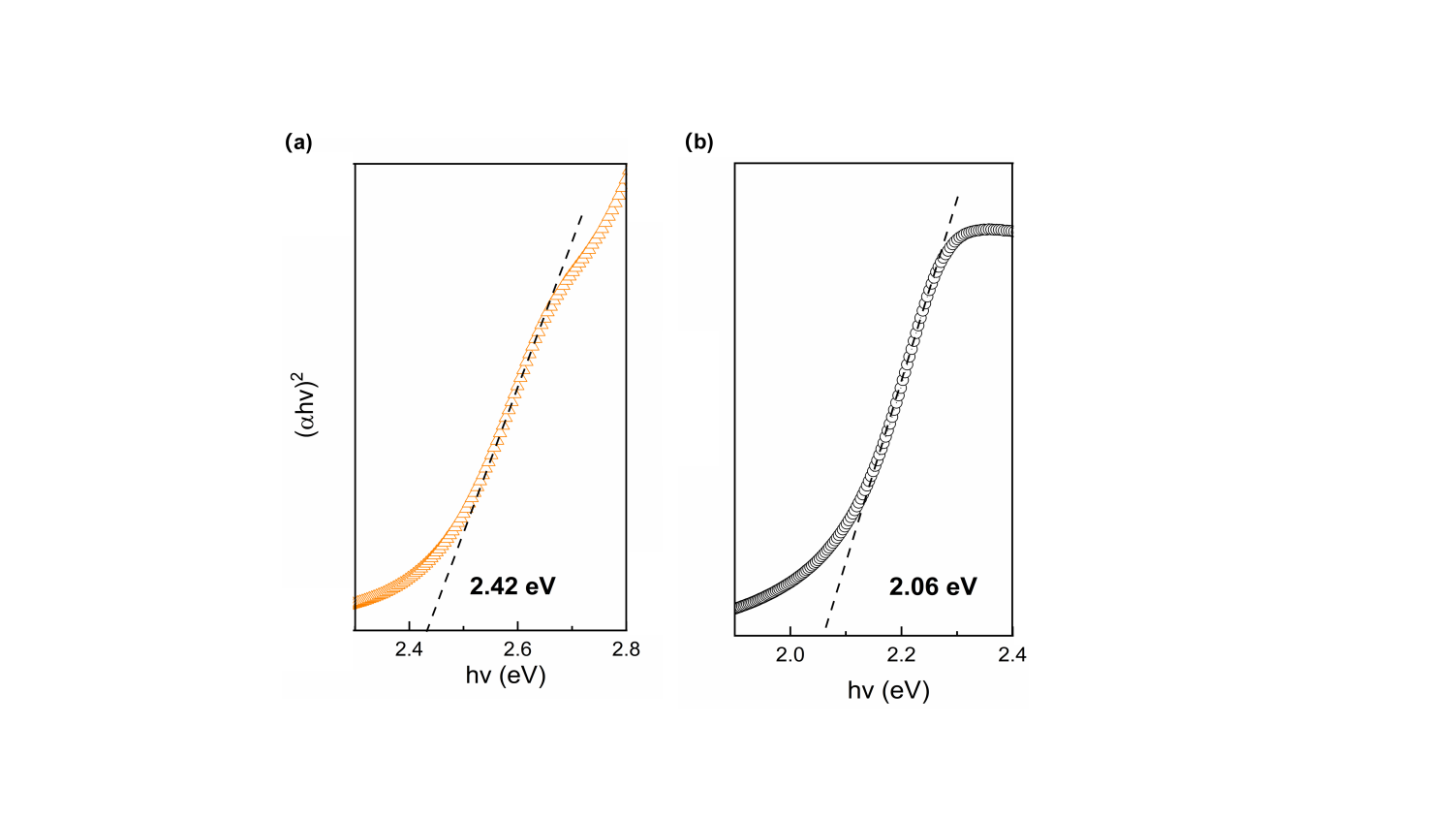


**Figure S2**. Bandgaps of Cs_3_Sb_2_I_9-x_Cl_x_ and MA_3_Sb_2_I_9-x_Cl_x_ films annealed at 100°C.


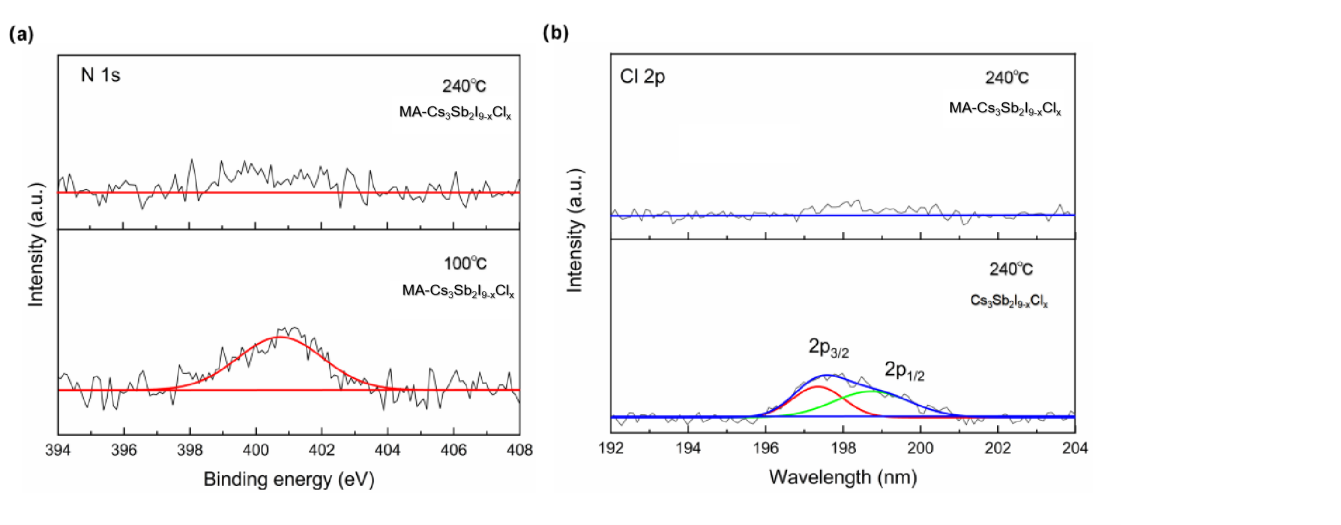


**Figure S3**. XPS spectra for (a) N 1s core levels of MA-Cs_3_Sb_2_I_9-x_Cl_x_ films annealed at 100°C and 240°C; (b) Cl 2p core levels of MA-Cs_3_Sb_2_I_9-x_Cl_x_ films annealed at 240°C.


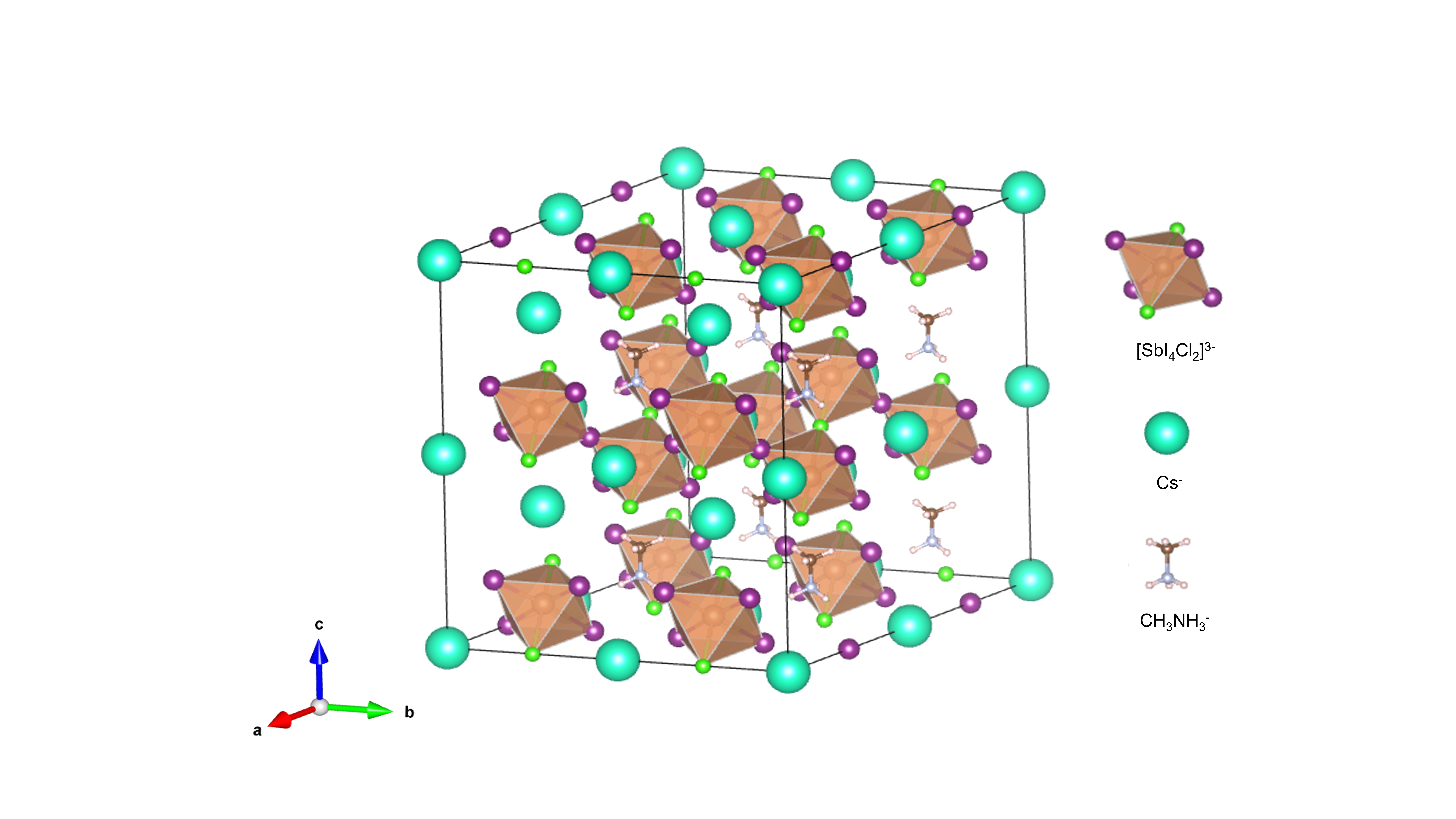


**Figure S4**. The 2×2×2 supercell of MACs_2_Sb_2_I_6_Cl_3_.


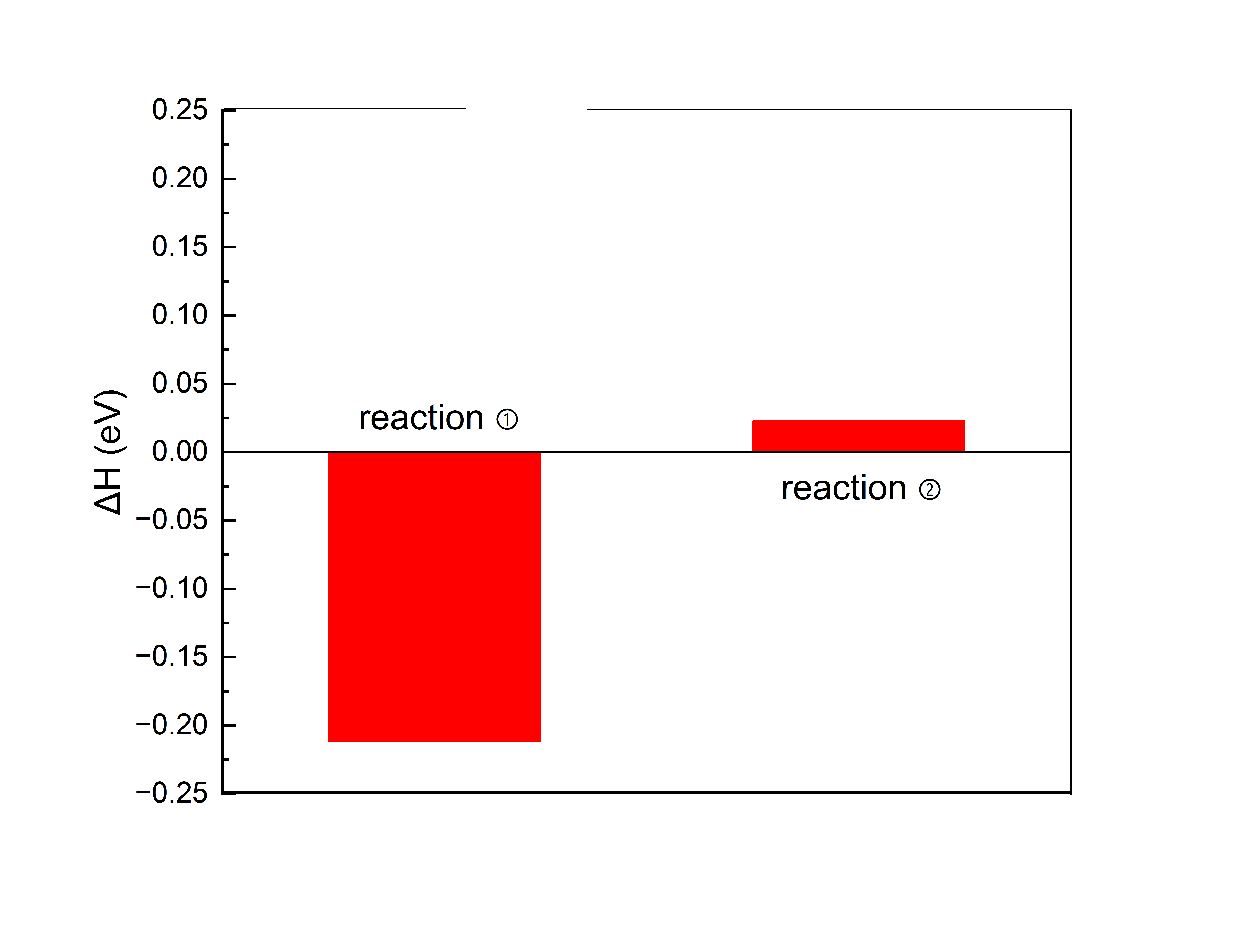


**Figure S5**. The calculated reaction enthalpy of the different reaction progresses.


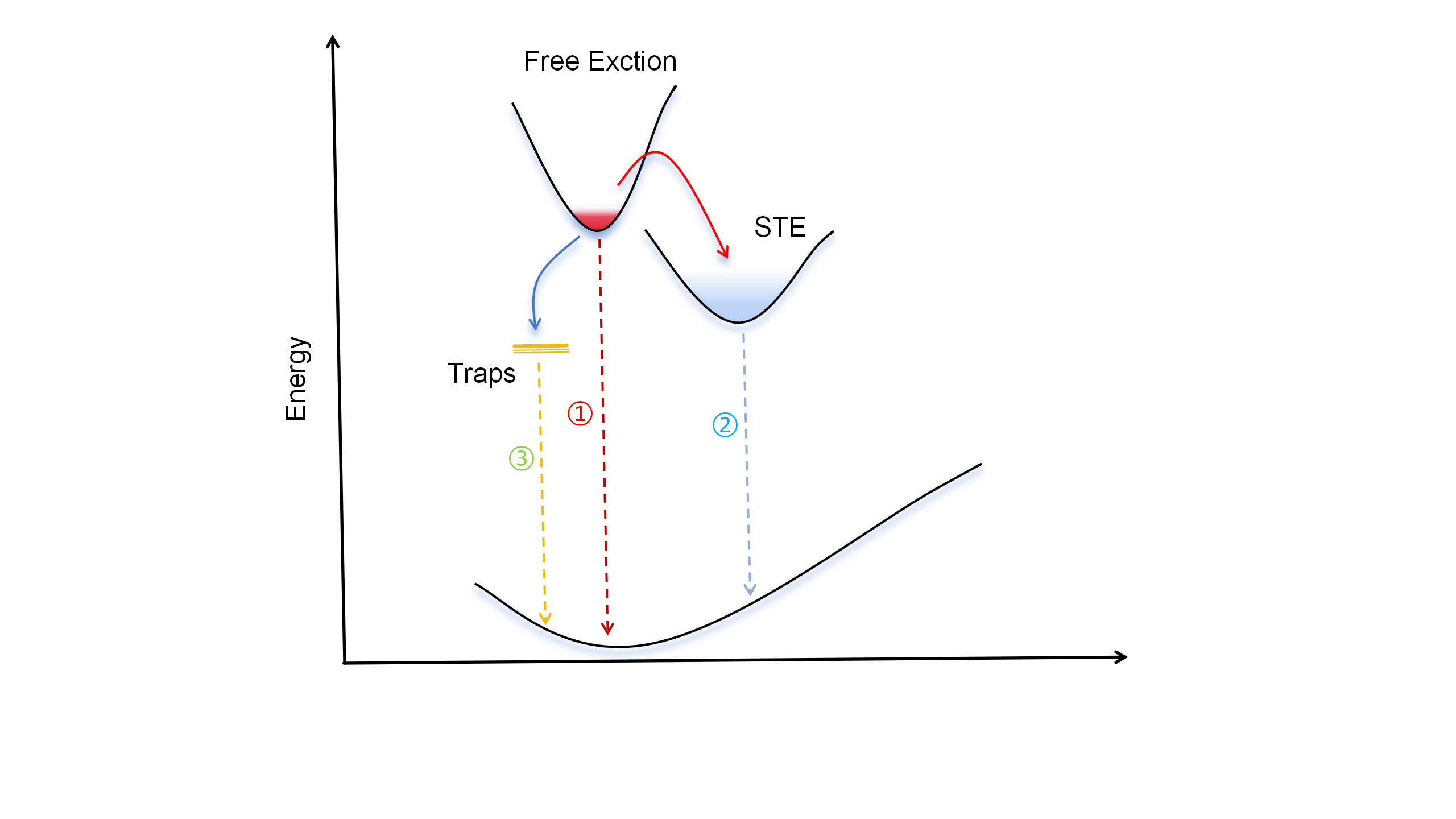


**Figure S6**. The schematic of photophysical process for PL spectra.


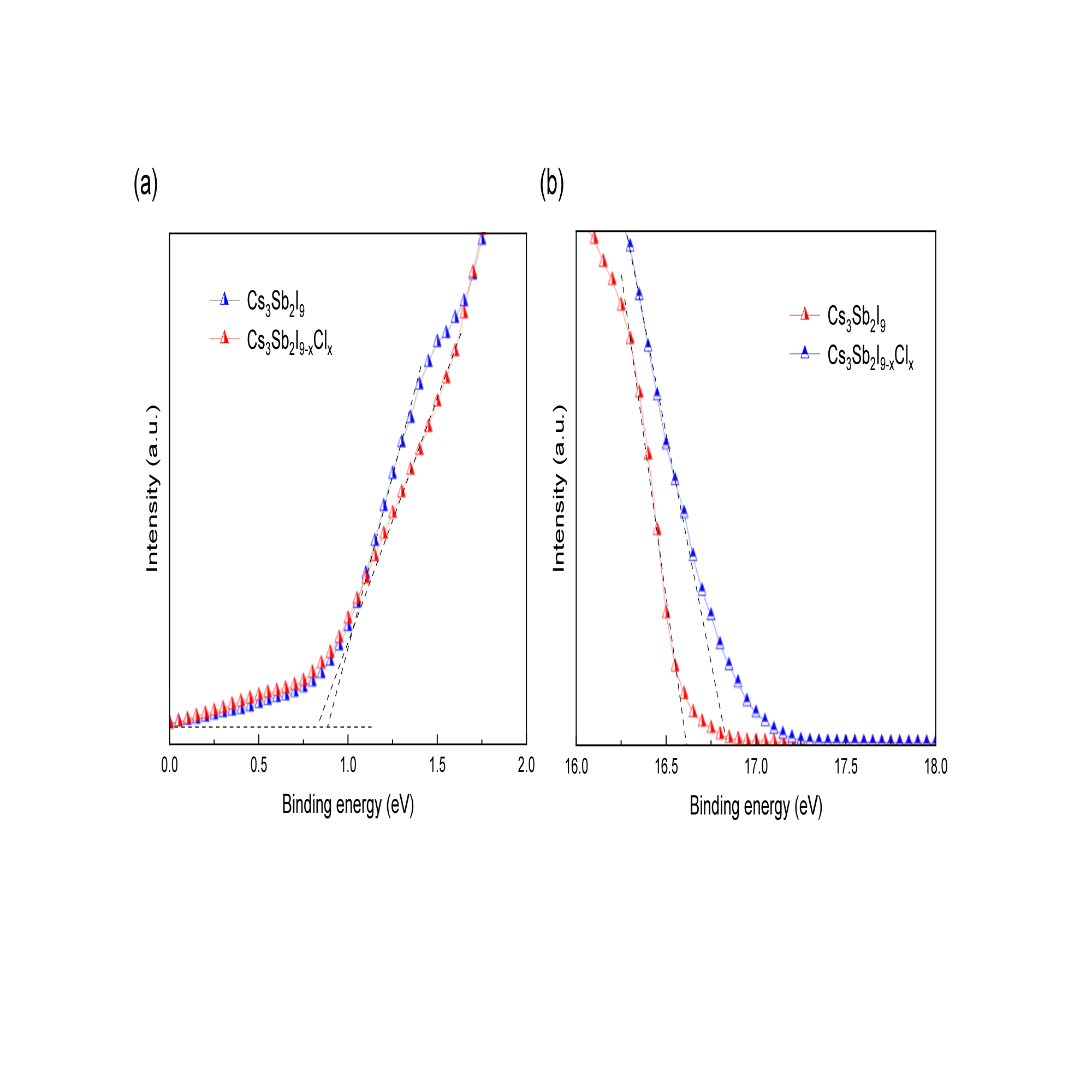


**Figure S7**. **(a)** Secondary electron cutoff edges and  **(b)** Fermi edges and of 2D-Cs_3_Sb_2_I_9-x_Cl_x_ and 2D-Cs_3_Sb_2_I_9_ films.


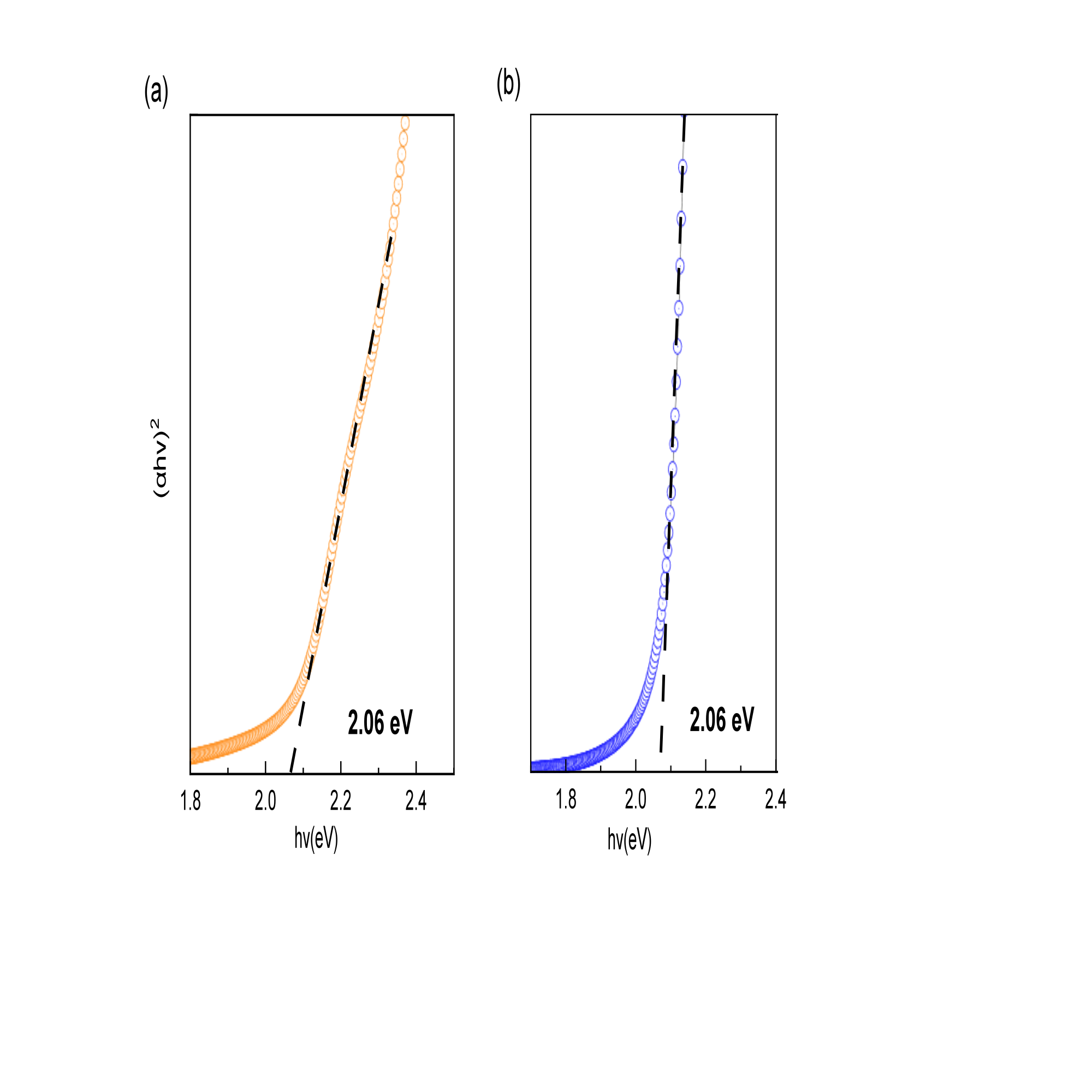


**Figure S8**. Bandgaps of (a) 2D-Cs_3_Sb_2_I_9-x_Cl_x_ and (b) 2D-Cs_3_Sb_2_I_9_ films.


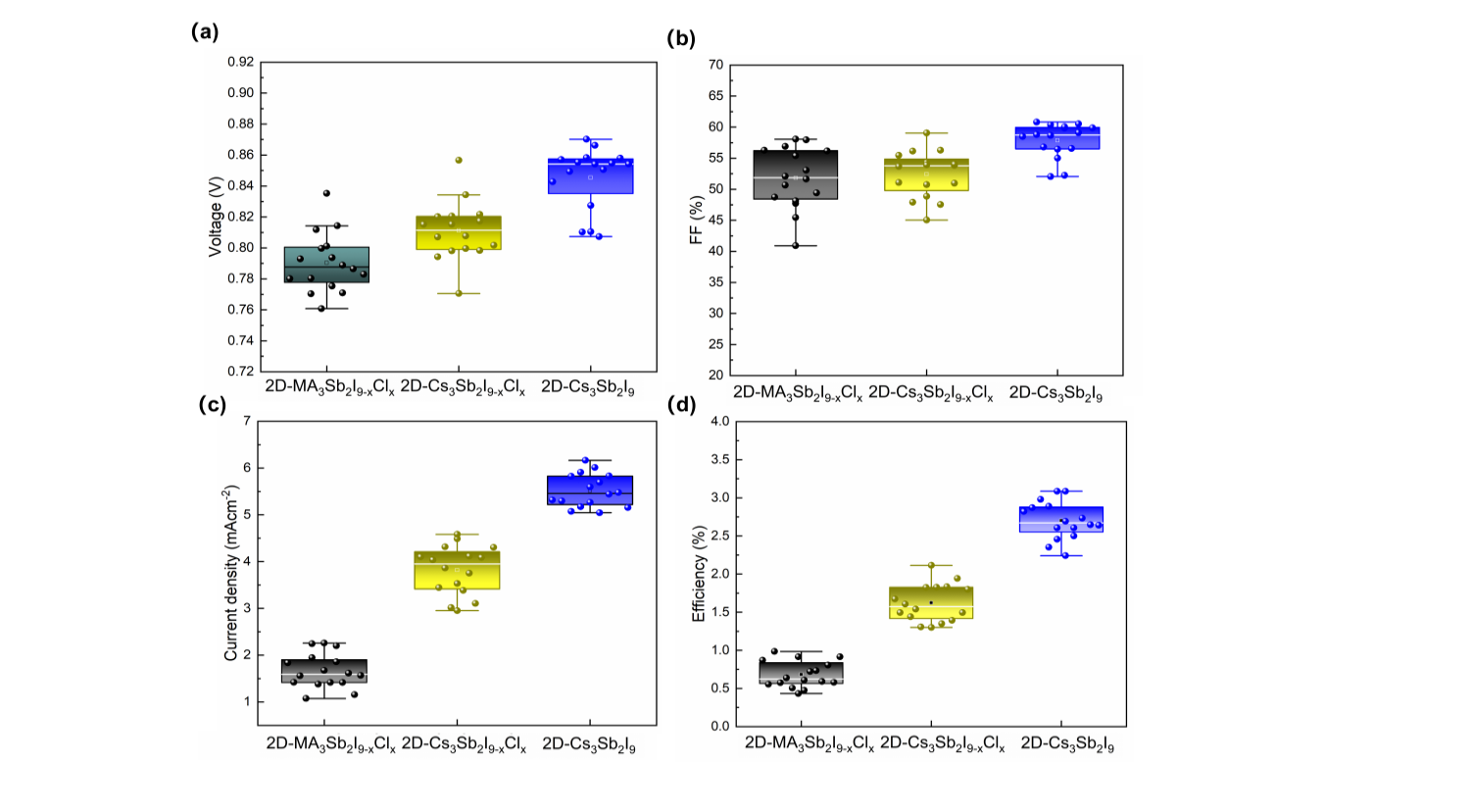


**Figure S9**. The statistic parameters of (a) *V_oc_*, (b) FF, (c) *J_sc_*, and (d) PCE of the devices of 2D-MA_3_Sb_2_I_9-x_Cl_x_, 2D-Cs_3_Sb_2_I_9-x_Cl_x_, and 2D-Cs_3_Sb_2_I_9_.


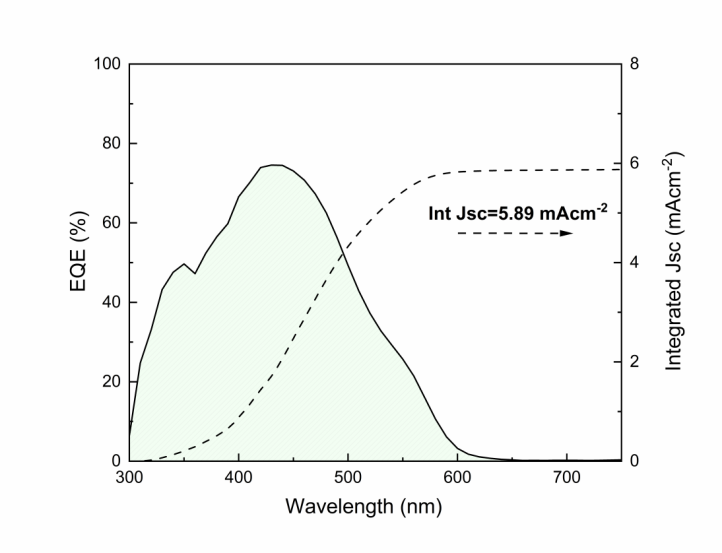


**Figure S10**. EQE spectrumof the 2D-Cs_3_Sb_2_I_9_ devices with F4TCNQ doped P3HT.


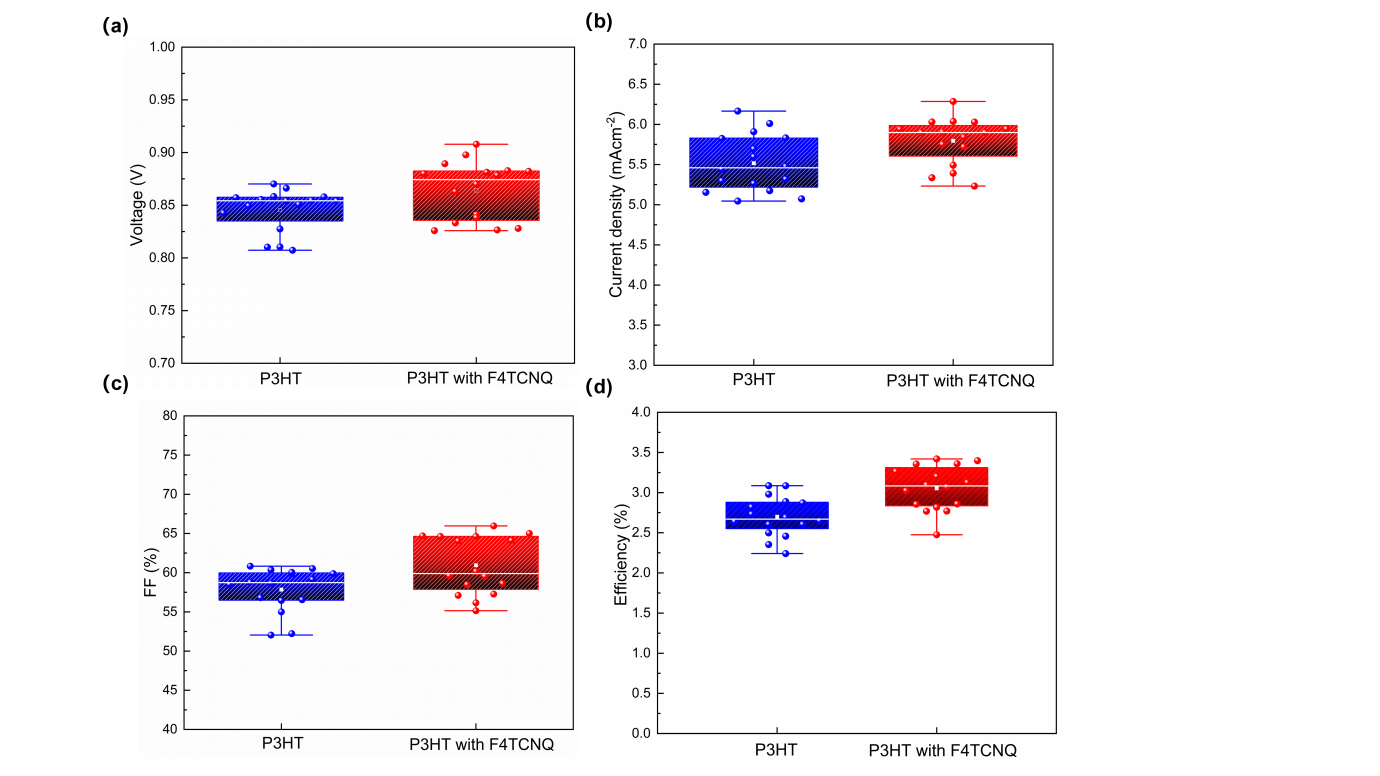


**Figure S11**. The statistic parameters of (a) *V_oc_*, (b) FF, (c) *J_sc_*, and (d) PCE of the 2D-Cs_3_Sb_2_I_9_ devices with P3HT and F4TCNQ.


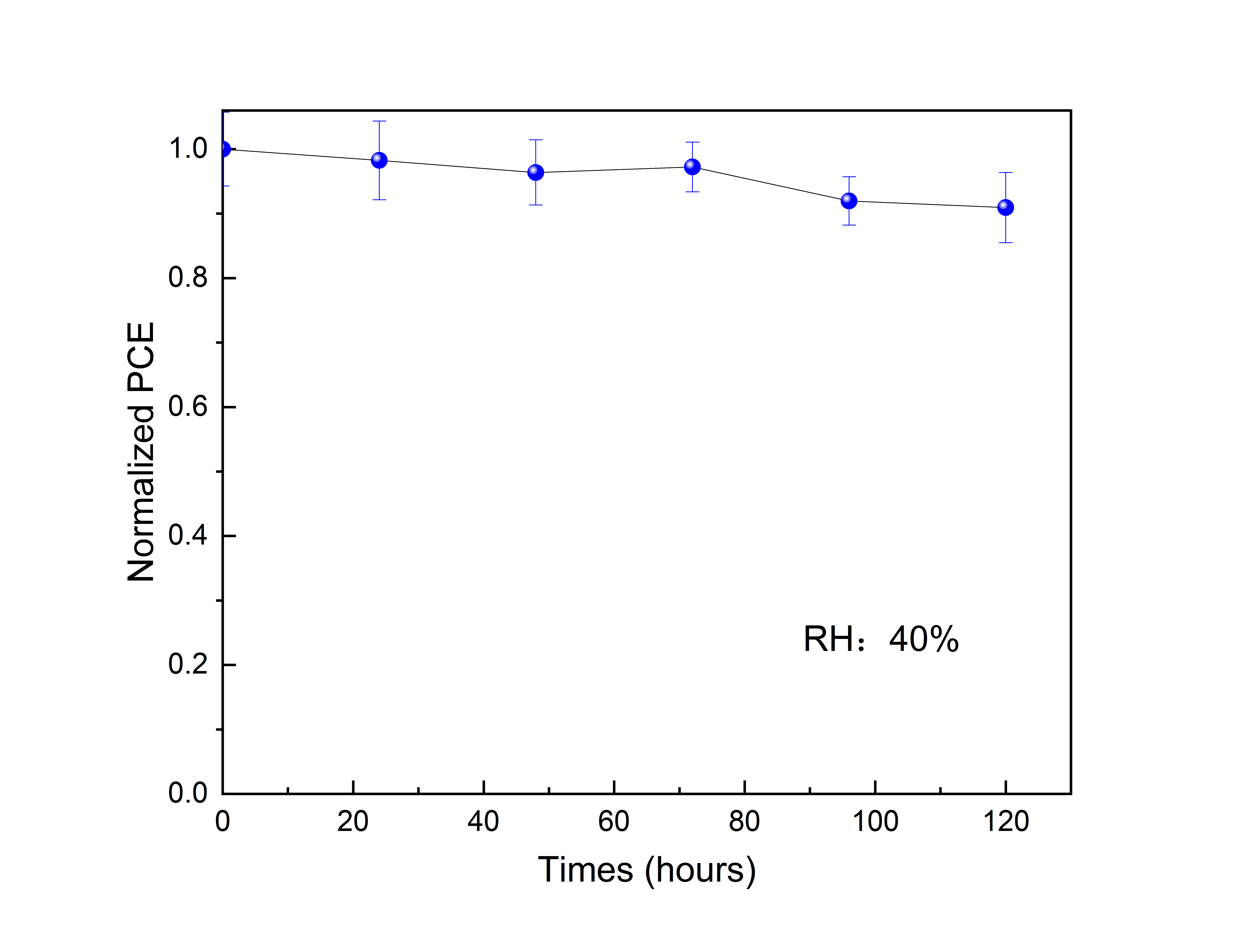


**Figure S12** .Long-term operational stability of unencapsulated 2D-Cs_3_Sb_2_I_9_ devices under ambient indoor conditions (RH ≈ 40%, room temperature).

**Table S1.** Raman peaks and corresponding vibrations of 0D-Cs_3_Sb_2_I_9_

| **Vibration** | **Symmetry** | **Description** | **Freq (cm^-1^)** |
| --- | --- | --- | --- |
| $\nu_{1}$ | $A_{1}^{'}$ | symmetric terminal Sb-I stretching | 172.6 |
| $\nu_{2}$ | $A_{1}^{1}$ | symmetric bridging Sb-I stretching | 111.5 |
| $\nu_{10}$ | $E^{'}$ | asymmetric terminal Sb-I stretching | 133.7 |
| $\nu_{15}$ | $E^{''}$ | asymmetric terminal Sb-I stretching | 148.2 |
| $\nu_{7}$ | $E^{''}$ | asymmetric bridging Sb-I stretching | 96.7 |
|  |  | Cs-[Sb_2_I_9_] bending modes | 66.9 |

**Table S2.** DFT calculated total energies of the corresponding compounds.

| **Materials** | **Total energies (eV)** |
| --- | --- |
| MA$\text{C}\text{s}_{2}\text{S}\text{b}_{\text{2}}\text{I}_{\text{6}}\text{Cl}_{\text{3}}$ | -77.3 |
| $\text{C}\text{s}_{\text{3}}\text{S}\text{b}_{\text{2}}\text{I}_{\text{6}}\text{Cl}_{\text{3}}$ | -40.56 |
| $\text{C}\text{s}_{\text{3}}\text{S}\text{b}_{\text{2}}\text{I}_{\text{9}}$ | -37.32 |
| $\text{ }\text{MAI}$ | -42.23 |
| $\text{ }\text{CsI}$ | -5.51 |
| $\text{MACl}$ | -43.38 |

**Table S3.** Fitting results of TA decays.

| **Sample** | **A_1_** | **t_1_ (ps)** | **A_2_** | **t_2_ (ps)** | **A_3_** | **t_3_ (ps)** | **t_1/e_ (ps)** |
| --- | --- | --- | --- | --- | --- | --- | --- |
| 2D-Cs_3_Sb_2_I_9-x_Cl_x_ | 0.91 | 2.01 | 0.36 | 23.12 | 0.09 | 1078.38 | 8.60 |
| 2D-Cs_3_Sb_2_I_9_ | 0.84 | 2.45 | 0.36 | 24.49 | 0.10 | 1737.63 | 9.19 |

**Table S4.** Comparison of the bond lengths of 2D-2D-Cs_3_Sb_2_I_6_Cl_3_ and 2D-Cs_3_Sb_2_I_9_ in the ground state and excited state.

| **2D-Cs_3_Sb_2_I_9_** | **Sb-I** | **Sb-I** | **Sb-I** | **Sb-I** | **Sb-I** | **Sb-I** | ***∆d*** |
| --- | --- | --- | --- | --- | --- | --- | --- |
| excited state | 3.140 | 3.140 | 3.140 | 2.958 | 2.958 | 2.958 | 0.898×10^-3^ |
| **2D-Cs_3_Sb_2_I_6_Cl_3_** | **Sb-I** | **Sb-I** | **Sb-Cl** | **Sb-I** | **Sb-I** | **Sb-Cl** | ***∆d*** |
| excited state | 3.157 | 3.157 | 2.908 | 3.061 | 3.061 | 2.506 | 5.752×10^-3^ |

**Table S5.** Photovoltaic performance of champion solar cells with different absorbance layers.

| **Absorber layer** | **V_OC_ [V]** | **J_SC_ [mAcm^-2^]** | **FF** | **PCE [%]** |
| --- | --- | --- | --- | --- |
| 2D-MA_3_Sb_2_I_9-x_Cl_x_ | 0.79 | 2.26 | 0.55 | 0.98 |
| 2D-Cs_3_Sb_2_I_9-x_Cl_x_ | 0.82 | 4.58 | 0.56 | 2.11 |
| 2D-Cs_3_Sb_2_I_9_ | 0.87 | 5.83 | 0.61 | 3.09 |

**Table S6.** Photovoltaic performance of champion solar cells with P3HT and F4TCNQ - doped P3HT.

| **Absorber layer** | **V_OC_ [V]** | **J_SC_ [mAcm^-2^]** | **FF** | **PCE [%]** |
| --- | --- | --- | --- | --- |
| P3HT | 0.87 | 5.83 | 0.61 | 3.09 |
| F4TCNQ-doped P3HT | 0.88 | 5.94 | 0.65 | 3.4 |

**Table S7.** A summary of the indoor photovoltaic performance parameters (1000lux,LED) for Sb/Bi based perovskite-inspired based solar cells.

| **Sample** | **Structure** | **PCE(%)** | **Ref** |
| --- | --- | --- | --- |
| Cs_3_Sb_2_Cl_x_I_9-x_ | FTO/TiO_2_/Sample/Poly-TPD/Au | 4.4% | [1] |
| FAMACs_3_Sb_2_Cl_x_I_9-x_ | FTO/TiO_2_/Sample/P3HT/Au | 6.37% | [2] |
| Cs_3_Sb_2_I_9_ | FTO/Nb_2_O_5_/Sample/P3HT/Carbon  FTO/TiO_2_/Sample/P3HT/AupP/P3HT/Au/layer/P3HT/Aulayer /Spiro-OMeTAD/Ag | 8.2% | **Our work** |
| (CH_3_NH_3_)Bi_2-x_In_x_I_9_ | FTO/TiO_2_/Sample/Spiro-OMeTAD/Ag | 5.9% | [3] |
| AgBiI_4_ | FTO/TiO_2_/Sample/ Spiro-OMeTAD/Au | 5.17% | [4] |
| Ag_2_BiI_5_ | ITO/SnO_2_/Sample/PTAA/Au | 5.02% | [5] |
| BiOI | ITO/NiO_x_/Sample/ZnO/Cr/Ag | 4% | [1] |

**Reference:**

[1] Y. Peng, T. N. Huq, J. Mei, L. Portilla, R. A. Jagt, L. G. Occhipinti, J. L. Macmanus Driscoll, R. L. Hoye, V. Pecunia, *Adv. Energy Mater.* **2021**, *11*, 2002761.

[2] N. Lamminen, G. K. Grandhi, F. Fasulo, A. Hiltunen, H. Pasanen, M. Liu, B. Al Anesi, A. Efimov, H. Ali Löytty, K. Lahtonen, *Adv. Energy Mater.* **2023**, *13*, 2203175.

[3] R. Kumar, H. Liu, S. A. Nabavi, M. S. Anyebe, S. Mahesh, H. Snaith, M. Bag, S. M. Jain, A*CS Appl. Electron.*Mat*er.* **2024**, *6*, 8360.

[4] I. Turkevych, S. Kazaoui, N. Shirakawa, N. Fukuda, Jpn. *Jpn. J. Appl. Phys.* **2021**, *60*, SCCE6.

[5] N. B. C. Guerrero, Z. Guo, N. Shibayama, A. K. Jena, T. Miyasaka, *ACS Appl. Energ. Mate*r. **2023**, 6, 11.
